# A Systematic Computational Analysis of Biosynthetic Gene Cluster Evolution: Lessons for Engineering Biosynthesis

**DOI:** 10.1371/journal.pcbi.1004016

**Published:** 2014-12-04

**Authors:** Marnix H. Medema, Peter Cimermancic, Andrej Sali, Eriko Takano, Michael A. Fischbach

**Affiliations:** 1 Department of Microbial Physiology, Groningen Biomolecular Sciences and Biotechnology Institute, University of Groningen, Groningen, The Netherlands; 2 Groningen Bioinformatics Centre, Groningen Biomolecular Sciences and Biotechnology Institute, University of Groningen, Groningen, The Netherlands; 3 Department of Bioengineering and Therapeutic Sciences, University of California, San Francisco, San Francisco, California, United States of America; 4 California Institute for Quantitative Biosciences, San Francisco, California, United States of America; 5 Department of Pharmaceutical Chemistry, University of California, San Francisco, San Francisco, California, United States of America; 6 Manchester Institute of Biotechnology, Faculty of Life Sciences, University of Manchester, Manchester, United Kingdom; Hellas, Greece

## Abstract

Bacterial secondary metabolites are widely used as antibiotics, anticancer drugs, insecticides and food additives. Attempts to engineer their biosynthetic gene clusters (BGCs) to produce unnatural metabolites with improved properties are often frustrated by the unpredictability and complexity of the enzymes that synthesize these molecules, suggesting that genetic changes within BGCs are limited by specific constraints. Here, by performing a systematic computational analysis of BGC evolution, we derive evidence for three findings that shed light on the ways in which, despite these constraints, nature successfully invents new molecules: 1) BGCs for complex molecules often evolve through the successive merger of smaller sub-clusters, which function as independent evolutionary entities. 2) An important subset of polyketide synthases and nonribosomal peptide synthetases evolve by concerted evolution, which generates sets of sequence-homogenized domains that may hold promise for engineering efforts since they exhibit a high degree of functional interoperability, 3) Individual BGC families evolve in distinct ways, suggesting that design strategies should take into account family-specific functional constraints. These findings suggest novel strategies for using synthetic biology to rationally engineer biosynthetic pathways.

## Author Summary

Bacterial secondary metabolites mediate a broad range of microbe-microbe and microbe-host interactions, and are widely used in human medicine, agriculture and manufacturing. Despite recent advances in synthetic biology, efforts to engineer their biosynthetic genes for the production of unnatural variants are frustrated by a high failure rate. In an effort to better understand what types of genetic changes are most likely to lead to successful improvements, we systematically analyzed the ways in which biosynthetic genes naturally evolve to generate new compounds. We show that large gene clusters appear to evolve through the merger of sub-clusters, which function independently, and are promising units for cluster engineering. Moreover, a subset of gene clusters evolve by concerted evolution, which generates sets of interoperable domains that may enable predictable domain swapping. Finally, many biosynthetic gene clusters evolve in family-specific modes that differ greatly from each other. Overall, this quantitative perspective on the ways in which gene clusters naturally evolve suggests novel strategies for using synthetic biology to engineer the production of unnatural metabolites.

## Introduction

Bacterial secondary metabolites are widely used as pharmaceutical, agricultural, and dietary agents. They consist of many classes of compounds including polyketides (PKs), nonribosomal peptides (NRPs), ribosomally synthesized and post-translationally modified peptides (RiPPs), terpenoids, saccharides, and a plethora of hybrids. The genetic basis for this rich molecular diversity can be found in biosynthetic gene clusters (BGCs), physically clustered groups of genes that encode the enzymatic pathways necessary to construct specific chemicals [1], [2].

The diversity of extant natural products and BGCs raises important questions about their evolutionary origin. These include the basic question of how Nature invents new molecules, and a series of applied questions relevant to biotechnology: for example, the evolutionary modularity of NRP and PK BGCs has long been seen as a feature that might allow large libraries of new compounds to be generated by mixing and matching their constituent domains and modules [3]. However, although there have been notable successes [4]–[6], the majority of combinatorially generated pathways appear to be nonfunctional [4]. More recently, advanced synthetic biology approaches to pathway engineering have been frustrated by the complexity and unpredictability of metabolic enzymes, particularly NRPSs and PKSs [7], [8]: unlike LEGO bricks, their constituent domains and modules do not ‘fit’ together universally, but only function effectively in specific pathway contexts.

Regardless of these apparent constraints to genetic change, Nature appears to have been quite successful at engineering biosynthetic pathways through the process of gene cluster evolution: even a conservative estimate suggests that the number of broad biosynthetic gene cluster families that have evolved exceeds 6,000 [8], most of which contain multiple BGCs that synthesize derivatives of a common scaffold. Hence, a detailed study of evolutionary patterns within various BGC families has the potential to offer a new inroad into effective BGC engineering, through mimicry of Nature's evolutionary design strategies.

So far, insights into the key principles underlying the evolution of BGC architectures and repertoires have been derived from limited case studies [9]–[13], which lack sufficient detail about the generality of the underlying mechanisms. Here, we systematically quantify the strategies that make evolution so successful at engineering BGC diversity. Through a detailed computational analysis of a recently generated dataset of 732 known and 10,724 predicted prokaryotic BGCs [8], we find that the rates of evolutionary events, such as insertions, deletions and duplications within BGCs, are much higher than those seen in comparable gene clusters involved in primary metabolism. Furthermore, distinct sub-clusters consisting of co-evolving genes appear to constitute relatively independent building blocks that play key roles in the evolution of larger BGCs encoding the biosynthesis of complex metabolites. Finally, BGC families encoding the production of polyketides and nonribosomal peptides evolve in family-specific modes, in many of which we observe an unexpectedly large role for concerted evolution [14], [15] driven by internal recombinations. Based on these observations, we offer several recommendations for establishing new modes of evolution-guided BGC engineering.

## Results/Discussion

### BGCs are rapidly evolving genomic entities

**Figure 1:**
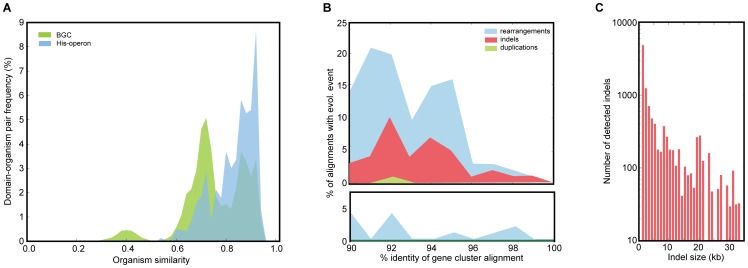
The rapid and dynamic evolution of BGCs differs from the evolution of ribosomal gene clusters and primary metabolism. **a**, Distributions of the best matching sequence homologs with respect to organism similarity (based on 16S rRNA) for predicted BGCs and histidine operons suggest significant differences in the ways they evolve. **b**, Number of detected rearrangements, indels and duplications plotted against the average percent identity in the aligned gene cluster pairs from which the events were deduced for predicted BGCs (top) and ribosomal gene clusters (bottom). Ribosomal gene clusters were selected for comparison based on their relatively large sizes (∼10–15 kb) compared to primary metabolic operons; to obtain a fair comparison with BGCs, only gene clusters of sizes 5–15 kb were taken into account. Counts are based on a systematic comparison of all gene clusters in our data set that share regions of >1000 bp with >70% identity, in which events were inferred from alignments of such 1000 bp blocks. Of the 10,096 BGC pairs meeting these criteria, 1,750 had a rearrangement, 1,140 had an indel, and 135 had a duplication, each of which were far more common than the corresponding evolutionary events in gene clusters encoding the translation apparatus. Interestingly, while indels and rearrangements could be detected in ∼16% and ∼19% of BGCs of all sizes, duplications are found far more commonly in gene clusters with sizes of >40 kb (7.6%) than in gene clusters with sizes of 10–20 kb (0.3%), suggesting a possible role for duplication and divergence in the evolution of large gene clusters. **c**, Size distribution of inserted/deleted fragments during recent gene cluster evolution, based on the indel analysis.

The large diversity of BGCs observed throughout the prokaryotic tree of life [8] suggests that BGCs evolve rapidly. Indeed, when we systematically quantified different evolutionary events by mutually comparing all gene clusters in our data set (**Table S1**), we found not only that they may have been transferred horizontally at high frequency (**Fig. 1a** **and Figure S1**), but also display exceptionally high rates of insertions, deletions, duplications and rearrangements (**Fig. 1b**). While the percentage of gene cluster pairs related by an indel is independent of gene cluster size, the distribution of indel sizes shows a long tail that includes 195 indels of 10 kb or more (**Fig. 1c**). As expected, these large indels are more commonly found in larger gene clusters, where they indicate either the merger of one gene cluster fragment with another or the loss of a gene cluster fragment from a larger cluster (see examples in **Figure S2**). Phylogenetic profiling [16] showed that many such BGC fragments – here termed sub-clusters – appear to evolve in a correlated fashion: 884 different motifs of adjacent Pfam domains (out of 7,641 found) were shown to co-evolve significantly more often than not (*P*<0.001), based on the χ^2^ test. These motifs comprise 591 different Pfam domains and have an average length of 5.3 domains (**Table S2**). As expected, they include many well-known and widely conserved motifs that appear to be linked to specific sub-functionalities of gene clusters, such as precursor biosynthesis, transport or synthesis of a specific chemical moiety, and motifs belonging to modular BGC architectures of NRPSs and PKSs (e.g., C-A-T and KS-AT-T [17]).

### Sub-cluster sharing enables evolutionary ‘recombineering’ of BGCs

**Figure 2:**
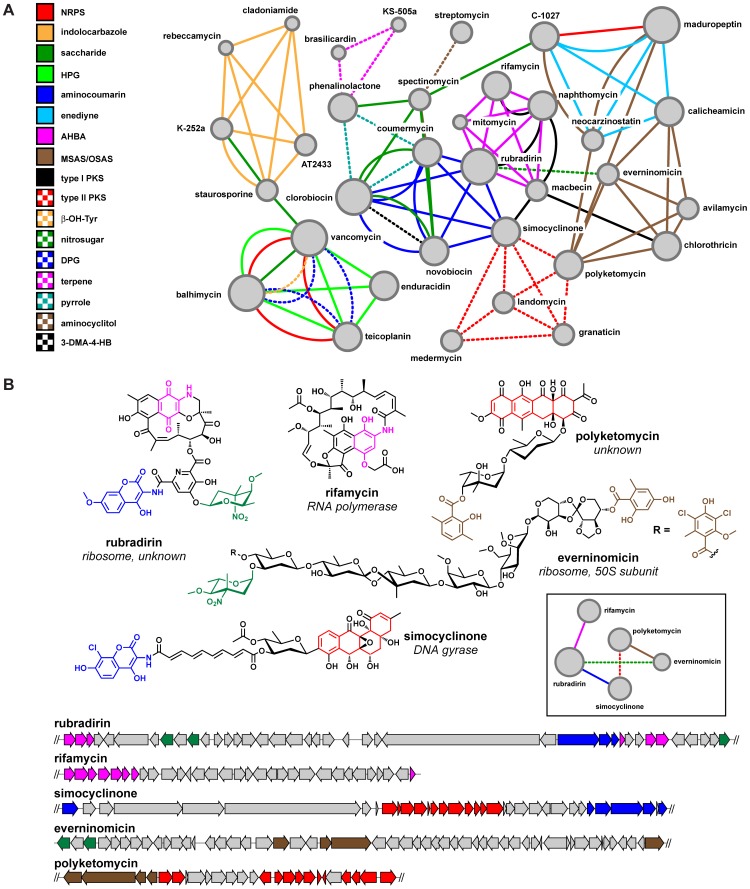
Complex BGC architectures evolve through new combinations of sub-clusters that are shared between multiple gene cluster types. **a**, Network of sub-clusters shared among 34 known BGCs. Nodes represent BGCs, and node size indicates the number of sub-clusters present in the gene cluster that are shared with other BGCs within the network. Edges represent shared sub-clusters, coded by color. The pattern of sharing indicates that many sub-clusters are regularly transferred between BGCs of different types. In the interpretation of this analysis, it should be kept in mind that in rare cases different biosynthetic routes (and hence, different sub-clusters) exist towards the same moiety. **b**, A sub-network from **a** showing the shared sub-clusters among the BGCs for rubradirin, rifamycin, simocyclinone, everninomicin, and polyketomycin, as well as the chemical moieties encoded by the sub-clusters.

Earlier evidence has suggested complex mosaic patterns of sub-cluster sharing for some BGCs, such as those involved in the production of glycopeptides [18]. To further explore the role of sub-cluster sharing in the evolution of BGCs, we manually compiled a set of 35 BGCs that are rich in sub-clusters that have a known connection with a specific chemical moiety. We then used this data set to construct a network in which the nodes represent BGCs and the edges denote a sub-cluster that a pair of BGCs has in common (**Fig. 2**). Three observations were particularly notable (**Fig. 2**). First, >60% of the coding capacity of some BGCs (e.g., those encoding vancomycin and rubradirin [19]) is composed of individually conserved sub-clusters (note that this is not entirely reflected in the depiction of the rubradirin gene cluster in **Fig. 2b**, where only those sub-clusters are highlighted that are shared with other depicted BGCs). This supports a “bricks and mortar” model of gene cluster evolution in which gene clusters are composed of large, modular “bricks” (sub-clusters) that encode key building blocks and individual genes (the “mortar”) that encode functions such as tailoring, regulation and transport. During evolution, both bricks and mortar (scaffold and tailoring) may remain the same, only the tailoring may change or the scaffold itself may change. Second, the same sub-cluster commonly appears in otherwise unrelated BGCs, and multiple unrelated sub-clusters can be found in a single parent gene cluster, indicating that sub-clusters are independent evolutionary entities. Third, sub-clusters are not static; they are loosely organized around a core set of genes, but gene gain/loss leads to chemical changes in the corresponding part structure: for example, gene clusters encoding molecules such as everninomicin [20], simocyclinone [21] and polyketomycin [22] have different variants of deoxysugar sub-clusters, which lead to subtle variations in the final chemical structures.

Although the complex patterns of sub-cluster sharing, in which various sub-clusters are shared between otherwise completely different gene clusters (**Fig. 2**), indicate that BGCs may evolve by the successive merger of sub-clusters, this does not mean that every case where sub-clusters are shared points to an independent sub-cluster transfer event. For example, the KS domains of the diverse range of ansamycin type I PKS BGCs that harbor AHBA sub-clusters are almost completely monophyletic (**Figure S3**), indicating that the macrolactam- and AHBA-producing sub-clusters have been co-evolving for a long time (instead of multiple independent AHBA sub-cluster acquisitions having occurred in different macrolactam-producing polyketide BGCs). Hence, the multi-hybrid rubradirin gene cluster might have arisen from a rifamycin-like ancestor (most rubradirin KS domains are monophyletic with rifamycin KS domains, see **Figure S3**) that already harbored the combination of a modular type I PKS sub-cluster and an AHBA biosynthesis sub-cluster, and which then acquired new sub-clusters for the biosynthesis of the aminocoumarin, 3,4-dihydroxydipicolinate and nitrosugar moieties (which are not found in any other closely related ansamycins). Contrary to the shared evolutionary histories of AHBA and ansamycin type I PKS sub-clusters, a clear example of sub-cluster transfer between BGCs of different types can be seen for 6-methylsalicylic acid (MSAS)/orsellinic acid (OSAS) sub-clusters, as inferred from a maximum-likelihood phylogenetic tree of MSAS/OSAS-producing iterative PKSs (**Figure S4**). The topology of this tree strongly indicates that MSAS/OSAS sub-clusters have largely evolved independent of the scaffold types of their parent gene clusters (**Figure S4**), and that they have been transferred between multiple types of BGCs during their evolutionary past. In conclusion, in the context of the bricks-and-mortar analogy, some bricks move around between different structures more often than others. Finally, we should note that there are also BGC families which evolve over long periods of time without major changes to the gene cluster architecture or the scaffold of the core molecule made: for example, the large family of over >1,000 aryl polyene BGCs that we described recently [8] has not undergone any major sub-cluster transfers, aside from the inclusion of the dialkylresorcinol sub-cluster in the BGCs from some CFB group bacteria. The products of many of these BGCs are likely to be entirely identical, while remaining differences between the molecules mostly concern differential tailoring of the same scaffold.

### Evolution from one scaffold to another

**Figure 3:**
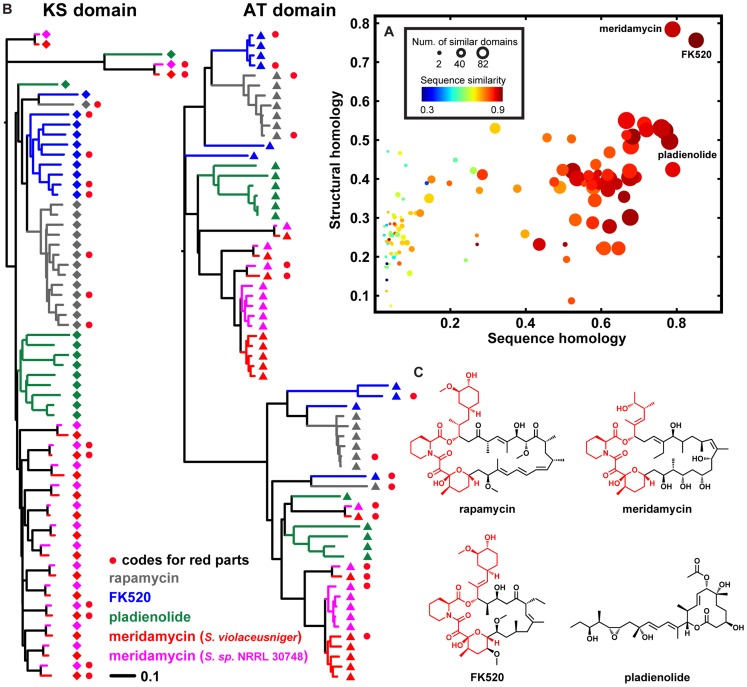
Unexpected evolutionary relationships within the rapamycin family. **a**, Distinct scaffolds produced by pathways from related BGCs. The scatter plot shows the relationship between the sequence homology of a pair of BGCs (x-axis) and the structural homology of their small molecule products (y-axis), compared to rapamycin and its BGC. Each circle represents a gene cluster and its small molecule product. Meridamycin and FK520 are closely related to rapamycin, as are their BGCs. While the pladienolide BGC is closely related to the rapamycin BGC, the structure of pladienolide itself is not very similar to that of rapamycin. In particular, pladienolide has a much smaller macrocycle and lacks shikimate- or pipecolate-derived moieties, and, as a result, binds to a distinct protein target. Structural similarity is estimated by the Tanimoto coefficient using linear-path fingerprints (FP2) from Open Babel [67], while sequence homology is represented as the Jaccard index defined on pairs of Pfam domains that share sequence identities within the top 10^th^ percentile of all-pair sequence identities. The number of domain pairs that share sequence identities within the top 10^th^ percentile and sequence identity of all domain pairs are shown as point sizes and colors, respectively. **b**, The role of concerted evolution in homogenizing domains within a BGC. Phylogenetic trees of KS and AT domains from the rapamycin, FK520, meridamycin, and pladienolide BGCs are shown (for detailed trees with accession numbers and bootstrap values, see **Figure S11**). The KS and AT sequences largely cluster into BGC-specific clades; for the AT domains, this is even the case for two different clusters encoding the same compound (meridamycin), showing the ability of concerted evolution to homogenize domains within a BGC. **c**, Chemical structures of rapamycin, meridamycin, FK520 and pladienolide. The sub-structure shared among rapamycin, meridamycin and FK520 is colored red, and the domains responsible for the biosynthesis of this sub-structure in each molecule are indicated with red circles in **b**.

Many chemical scaffold types of secondary metabolite classes are quite distinct, which raises the question of how BGC families encoding the synthesis of distinct scaffolds are related. To assess this question, we calculated the proportion and similarity of Pfam domains shared between all pairs of BGCs within our data set of 732 known gene clusters using multiple sequence alignments for each Pfam domain (**Fig. 3**) and looked specifically for close homologues of BGCs just outside their immediate family. Even though of course sequence similarity alone does not provide conclusive evidence on evolutionary histories, the analysis did suggest that unexpected evolutionary connections might exist between natural products of different scaffold types.

For example, the *Streptomyces* gene cluster encoding the lipopeptide antibiotic daptomycin [23] is surprisingly similar to *Mycobacterium* glycopeptidolipid (GPL) gene clusters [24] (**Figure S5**). When we performed a more in-depth analysis through a phylogenetic analysis of condensation domains, we indeed found that GPL domains consistently cluster together with domains from the NRPSs that synthesize daptomycin (**Figure S6**). Although both daptomycin and the GPLs are lipopeptides, the *Mycobacterium* GPLs are shorter (tetrapeptide vs. tridecapeptide), cell-wall-associated rather than diffusible, linear rather than cyclic, and originate from an actinomycete genus that is not closely related to *Streptomyces*.

Likewise, one of the strongest matches for the gene cluster encoding the immunosuppressant rapamycin [25], apart from the closely related FK520 [26] and meridamycin [27], [28] BGCs, was the gene cluster for pladienolide [29], a polyketide of unrelated structure with a distinct biological activity (inhibition of the splicing factor SF3b instead of TOR). Strikingly, based on phylogenetic trees of their constituent ketosynthase (KS) and acyltransferase (AT) domains, the meridamycin gene cluster is more closely related to the pladienolide BGC than to those encoding rapamycin and FK520, the molecules to which it is often compared (**Fig. 3**). These examples suggest that closely related sets of protein domains can be reconfigured by evolution to yield a new scaffold that is chemically and biologically distinct.

### Concerted evolution in the rapamycin family

The phylogenetic trees of KS and AT domains from our data set of known BGCs revealed another unexpected finding: in spite of the structural similarity of rapamycin and FK520, 63% of the constituent domains of their polyketide synthases (PKSs) cluster into entirely separate clades (**Fig. 3b**, see also **Figure S8** which shows that relevant bootstrap values are almost all above 90). Even more remarkably, 14 out of 16 domains responsible for the biosynthesis of the sub-structure shared between these two molecules (shown in red in **Fig. 3c**) do not cluster together with the corresponding domain from the assembly line for the other molecule. This pattern of homology is consistent with a phenomenon called ‘concerted evolution’, the homogenization of DNA sequences within a given repetitive family caused by high rates of internal recombination [14], [15]. Given the similar sizes and architectures of the gene clusters and the structural similarity of their products, this is a much more parsimonious explanation for the patterns observed than convergent evolution of multiple similar gene clusters through successive duplication of an ancestral single-module PKS. Notably, previous phylogenetic analyses of PKS domains have also observed BGC-specific clades of PKS domains [10], [30], but not to the extent observed here for such closely related gene clusters: the fact that such a strong pattern is even observed for the AT domains of two different gene clusters that encode the same molecule [27], [28], meridamycin, shows that the underlying process may operate on very short time scales, and that recombination can remove almost all traces of independent evolution of these PKS modules. In the case of the rapamycin family, recombinations are likely to occur neutrally and have no effect on the structure of the small molecule product (rapamycin, meridamycin and FK520), whereas in other cases, single crossovers within or between gene clusters may dramatically change the modular architecture of a synthase [30]. Near-neutral changes brought about by gene conversion may occur at higher rates for some domains or domain types than for others: in the meridamycin gene clusters, no signs of gene conversion could (yet) be observed for KS domains, even though gene conversion manifested itself clearly when comparing the meridamycin clusters with those encoding rapamycin, FK520 and pladienolide. On the contrary, AT domain gene conversion was widespread even between the two meridamycin gene clusters. We speculate that for these BGCs, gene conversion events get fixated in the population at lower rates for KS domains because not all KS sequences work equally well for different polyketide chain lengths that occur at different points of the assembly line, so that the changes brought about by a conversion event are less neutral than for AT domains. Mapping of rapamycin family PKS sequence mutations onto the 3D structure of an AT- and KS-containing protein further supports this hypothesis (**Figure S7a**), showing widespread sequence variability at almost every position in the AT domains, except for the residues near the substrate binding site (**Figure S7b**). Mutations in KS domains, on the other hand, are mostly restricted to the regions in vicinity (around the core) of the substrate-binding site and the dimerization interface (**Figure S7c**), suggesting their importance in influencing substrate selectivity.

### An unexpectedly large role for concerted evolution

**Figure 4:**
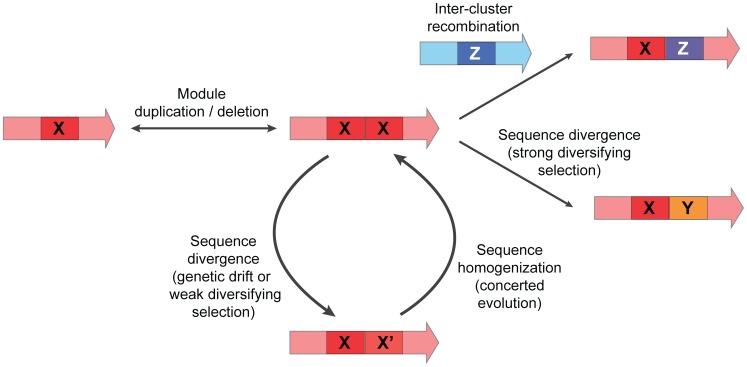
Qualitative model for the evolution of NRPS/PKS domains. After modules are duplicated, they may get ‘trapped’ in a cycle in which small sequence divergences are counterbalanced by internal recombinations that drive concerted evolution. Through strong diversifying selection (or sufficient drift), domains may break out of this cycle towards domain sequences that are protected from concerted evolution by functional divergence and subsequent stabilizing selection on the new function, or by reduced internal recombination rates due to larger sequence differences between the domains. The abovementioned sequence divergence may occur through cumulative mutation or through recombination with other gene clusters (or other modules within the same gene cluster).

Concerted evolution is not peculiar to the rapamycin family (**Figure S8**). For the gene clusters encoding the biosynthesis of the mutually closely related macrolides erythromycin [31], oleandomycin [32] and pikromycin [33], BGC-specific branching appeared to occur for both KS and AT domains, similar to the pattern for rapamycin, FK520, meridamycin and pladienolide. However, for the ansamycin antibiotics macbecin [34], geldanamycin [35] and herbimycin [36], and the antifungals pimaricin [37], nystatin [38] and amphotericin [39], BGC-specific branching occurs only for AT domains, and not for KS domains. Finally, corroborating earlier observations [40], domains from the trans-AT PKS gene clusters encoding pederin [41] and psymberin [42] do not show any BGC-specific branching at all. We observed that certain NRPS gene clusters also show signs of concerted evolution: a clear BGC-specific branching pattern pointing to concerted evolution can be seen for the A domains and most of the C domains of the gene clusters encoding the biosynthesis of the closely related calcium-dependent lipopeptides daptomycin [23], A54145 [43] and CDA [44]. However, the glycopeptide gene clusters encoding the biosynthesis of balhimycin [45], teicoplanin [46] and A40926 [47] showed no such pattern at all: almost all domains cluster in groups corresponding to domains in the same positions in the assembly line. Collectively, these observations suggest that concerted evolution is a key mechanism driving the evolution of NRPS and PKS gene sequences, but the extent to which it happens depends on family-specific functional constraints as well as on the presence of other evolutionary forces acting upon a gene cluster. Our qualitative model of PKS/NRPS evolution (**Fig. 4**), which summarizes the interplay of concerted evolution with other evolutionary mechanisms, is relevant to PKS/NRPS engineering efforts: the highly homologous sets of domains generated by concerted evolution are more likely to be mutually interoperable than domain sets chosen at random, and might therefore be attractive building blocks for synthetic biological engineering of biosynthetic pathways.

### Distinct mechanisms of PKS/NRPS BGC evolution

**Figure 5:**
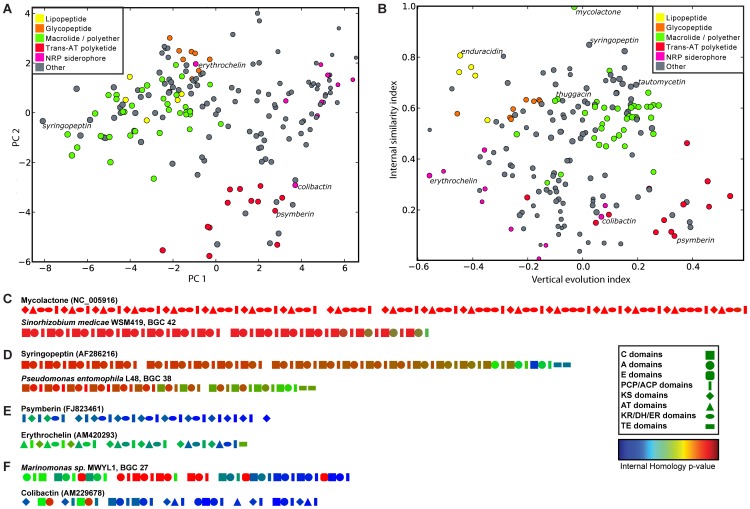
Diverse and distinct modes of evolution for PKS and NRPS BGCs. **a**, Scatter plot showing the first two principal components resulting from a PCA analysis of different evolutionary characteristics of BGCs encoding different classes of NRPs and PKs. The first two principal components describe 63% of the variance. BGCs encoding members of the same family (e.g., lipopeptides, glycopeptides or macrolides) tend to cluster together, suggesting that their family members evolve in similar ways, while different families cluster apart from each other, suggesting distinct modes of evolution. Colors indicate distinct classes of BGCs. **b**, Scatter plot showing two features of BGCs – internal similarity index and vertical evolution index – that, of the 25 measured features, underlie most of the variation. The internal similarity index indicates how similar domains in a BGC are to other domains within the same BGC. The vertical evolution index indicates how closely related a BGC is to the BGCs harboring the closest relatives of its constituent domains (see Methods for more details). Colors indicate distinct classes of BGCs, as in panel **a**. **c–f**, Domain architecture plots of PKSs and NRPSs show distinct modes of evolution: **c**, Internal duplication with concerted evolution; **d**, N-terminal additions by module duplication and recombination; **e**, domain swapping with other BGCs; and **f**, mixed evolution. Geometric shapes indicate domain types (see legend); domain colors indicate the internal homology p-value of each domain to its closest relative within the same gene cluster, within the total distribution of all similarities between domains of the same type in the entire data set: hence, domains colored red are most similar, while domains colored blue are most dissimilar.

To understand more generally how PKS and NRPS BGCs evolve, we set out to measure the contributions of concerted evolution, duplication, and divergence to the evolution of all multimodular PKS and NRPS BGCs in both our known and predicted BGC data sets. We first collected and quantified 25 different features describing the nature of gene cluster sequences and the relationships among their constituent domains (see methods for details). A principal component analysis (PCA) and hierarchical clustering using these features can distinguish many of the well-known gene cluster families from our data set of known BGCs (**Figure S9**, **Fig. 5a**). Two features in particular, the ‘internal similarity index’ and the ‘vertical evolution index’, explain much of the variation in terms of the modes of evolution of different classes of gene clusters (**Fig. 5b**). At the level of individual domains, we find that there are four primary mechanisms by which NRPS and PKS BGCs evolve (**Fig. 5c–f** **, Figure S10**). Firstly, gene clusters encoding glycopeptides, calcium-dependent lipopeptides and macrolides/polyethers appear to be most repetitive, pointing to a history of module duplications and/or a prominent influence of concerted evolution. The syringopeptin NRPS [48] and mycolactone PKS [49] are extreme examples of this: both are likely to have evolved recently by subsequent module duplications and concerted evolution. Secondly, we sometimes observed gradients of the internal homology *p*-values from the N- to C-termini of large synthases, suggesting that some gene clusters evolve to encode the synthesis of larger molecules by iterative duplication of their most N-terminal module, would have the effect of extending an intermediate NRP or PK by the addition of a new starter unit. Thirdly, a group of BGCs including the ones that encode the polyketides psymberin [42] and erythrochelin [50] show a ‘vertical’ type of evolution, in which the domains appear to evolve independently, with perhaps occasional domain swapping with related gene clusters, as has been suggested previously [40]. Finally, there are many gene clusters showing a ‘mixed’ mode of evolution, in which one or more of the above mechanisms are combined. For example, NRP siderophore gene clusters show some signs of internal recombinations, but at the same time many domains show no high mutual similarity. Like the trans-AT PKS gene clusters, they seem to have a higher tendency to recruit domains from dissimilar gene clusters. This recruitment over larger evolutionary distances appears to be a general feature of NRPS gene clusters as opposed to PKS gene clusters, and might be related to the wider range of possible substrates for NRPSs, which often require BGC-specific sub-pathways for the synthesis of a dedicated monomer [51].

### Birth and death of biosynthetic gene clusters during evolution

The observation of so many different evolutionary mechanisms of gene cluster evolution begs the question which circumstances lead to the birth and death of BGCs over evolutionary time. Are all BGCs that are detected bioinformatically also still intact and functional, or might many of them have degenerated and entered a nonfunctional state? The absence or presence of nonfunctional genetic units (e.g., pseudogenes or pseudo-gene-clusters) is largely governed by the evolutionary population dynamics of the species. Many bacteria live in large effective population sizes and have relatively short generation times, leading to very strong purifying selection and, consequently, rigorous genome streamlining [52]. Hence, BGCs that become nonfunctional will be quickly lost in such organisms if they do not provide any evolutionary advantage. Notably, some bacteria in fact occur in smaller population sizes and/or regularly go through population bottlenecks, leading to altogether different evolutionary dynamics [53]: in such cases, a range of pseudogenized gene clusters can sometimes still be observed that have not been purged from the genome yet [54]. On the whole, however, these appear to be rather the exception than the rule [55].

Concerning the birth of new gene cluster architectures, large effective population sizes and short generation times also suggest that BGC modifications should immediately confer an evolutionary advantage in order to be maintained; on the other hand, frequent changes in population size may affect the probability of mutations to be fixated in the population [56]. Alternatively, neutral mutations could hitchhike with strongly adaptive mutations within or close to the same gene cluster. Concerning the physical growth of gene clusters, it should be noted that new enzymes may already be recruited to a biosynthetic pathway before their genes are physically recruited to the gene cluster, and such an addition to a pathway could evolve through, e.g., positive selection acting on promiscuous enzyme activities or substrate specificities [57]. The precise reason for and evolutionary mechanism of clustering of biosynthetic genes in bacteria itself is still largely an unanswered question [58].

### Implications for biosynthetic engineering

Our analysis of BGC evolution will enable new approaches to BGC engineering informed by the mechanisms by which BGCs evolve naturally. Our results suggest that efforts to engineer the biosynthesis of unnatural natural products could be more successful by observing the modes by which specific BGC classes evolve in nature.

For example, conglomerate molecules consisting of multiple different chemical moieties could be designed by engineering BGCs consisting of novel combinations of sub-clusters. Such an effort could be guided by information taken from evolutionary comparisons, which would offer suggestions about which sub-clusters are most likely to function together, based on how often evolution has successfully forged combinations between them.

Furthermore, our evolutionary analysis of NRPS and PKS gene clusters suggests that concerted evolution has created sets of domains within gene clusters that are highly homologous. These domain sets are more likely to be mutually interoperable than domain sets chosen at random, and might therefore be of great utility in future engineering efforts.

Also, evolutionary strategies towards generating larger and more complex compounds could be mimicked by N-terminally extending certain types of NRPS/PKS gene clusters by duplicating and then carefully modifying the first assembly-line module.

Overall, in combination with new synthetic biology techniques that may soon enable the rapid assembly of thousands of clusters from a common set of parts [59]–[61], our results suggest a new approach for re-engaging gene cluster engineering in a manner informed by the mechanisms by which gene clusters have naturally evolved.

## Methods

### Comparison of HGT with primary metabolism

To remove highly similar genomes from these analyses, we used the AMPHORA [62] (August 10th, 2010) dataset, which contains gene sequences from 562 organisms for 30 universally conserved genes. Genes from these organisms were compared using sequence identities based on MUSCLE [63] multiple sequence alignments. This resulted in 30 distances between each pair of organisms. The distributions of distances of all pairs were tested for normality using a Shapiro-Wilk test. An organism distance map was then built with distances defined as the mean distances of AMPHORA genes. The resulting distance map was clustered using default settings in MCL [64], and only one member of each cluster was kept for further analyses. This left us with total of 408 organisms.

To search for histidine and tryptophan biosynthetic operons, we modified ClusterFinder [8]. Pfam [65] IDs associated with the histidine biosynthesis pathway (PF00475, PF00815, PF01174, PF01502, PF01634, PF04864, PF08029, and PF08645) or with the tryptophan biosynthesis pathway (PF00218, PF00290, PF00465, PF00697, PF01220, PF01264, PF01487, PF04715, and PF08501) were acquired from JGI IMG [66]. Trp or His operons were defined as gene clusters containing at least one of these domains with a probability >0.5 and containing at least two of the domains in total. Among 408 organisms searched, 350 His and 288 Trp biosynthesis operons were identified in 271 and 248 different organisms, respectively. The average number of domains per predicted gene cluster were 2.9 and 3.1, respectively.

Best matching sequence homologs of a query protein domain from a biosynthetic or primary metabolic gene cluster were obtained using MUSCLE [63] multiple sequence alignments. The distance between the organism containing the query protein domain and the organism with the best matching sequence homolog was determined based on 16S rRNA sequence similarity. Best matching sequence homologs of all protein domains that are in Pfam are included in the organism similarity histograms (**Fig. 1a**).

### Phylogenetic profiling

For each BGC, a two-dimensional array of the size corresponding to the numbers of consecutive protein domains that are in Pfam database (rows) and 408 selected organisms (columns) (see “Comparison of HGT with primary metabolism”) was created. The cells in the array consisted of sequence identities between a given domain from a BGC and the most homologous domain (which is also predicted as part of a BGC) from a given organism. Next, we calculated a Pearson product-moment correlation coefficient (correlation coefficient) for each possible pair of protein domains (rows), resulting into a new matrix, a correlation matrix, of the size corresponding to the number of protein domains (rows from the initial array) in both dimensions. To take rearrangements into account, we reordered rows and columns of the correlation matrix based on hierarchical clustering of the correlation matrix in both dimensions. We then parsed linear motifs that are likely to evolve in a correlated fashion by selecting consecutive pairs of domains in this reordered correlation matrix (consecutive fields on the first offset diagonal) with correlation coefficient >0.5. The analysis was repeated by setting the correlation coefficient cutoff to >0.65 and >0.8. Each motif was divided into all possible sub-motifs of sizes between 2 domains and the total number of domains in a motif. To determine the significance of a (sub)motif occurrence, we next compared the number of (sub)motif occurrences to the number of all possible (sub)motif occurrences in all BGCs that did not pass the correlation coefficient cutoff. Pearson's χ^2^ test with Bonferroni correction was applied to test for statistical significance, with the null hypothesis stating that the two values are equal.

### Analysis of recent evolution of BGCs

We performed an all-versus-all alignment of nucleotide sequences of known and predicted BGCs using the blastn algorithm. Gene cluster sequences were divided into blocks of 1 kb, and then mapped to the most homologous blocks from other gene clusters, as well as from the same gene cluster (to test for genomic duplications). 56% of the blocks (118,320 out of 212,176) did not map to any homologous regions in the same or other BGCs with >70% identity. Evolutionary events (insertions/deletions, duplications and rearrangements) were detected by a custom-made Python script (**Data S1**) comparing each alignment of two-gene clusters having at least three matching blocks with >70% identity. Rearrangements were defined as an identified difference in the order of 1-kb blocks in an otherwise conserved (piece of) gene cluster, such as when A1-A2-A3-A4-A5 matches to B1-B4-B3-B2-B5 in an alignment of two BGCs A and B. Indels were defined as 1-kb blocks present in one gene cluster but not in the other gene cluster, such as when A1-A2-A3-A4-A5-A6 matches to B1-B2-B5-B6 in an alignment. To make these inferences more reliable, a constraint was used that the flanking regions (of size > = 2 kb) of each indel breakpoint must be homologous between query and hit gene cluster, and the block order must be conserved between them. Finally, duplications were defined as 1-kb blocks that had the best hit towards another block in its own gene cluster, and having a higher copy number in one gene cluster than in the other, such as when A1-A2-A3-A2-A3-A4-A5 aligns to B1-B2-B3-B4-B5, while the mutual sequence identity between the A2 and A3 pairs is higher than between any of the A2/A3 blocks and B2 or B3.

### Comparison of sequence vs. structural similarity of gene clusters and their products

For a given BGC pair, we first calculated sequence identities between all Pfam domain pairs of each Pfam ID, using MUSCLE [63] multiple sequence alignments. A BGC sequence similarity index was defined as the Jaccard index with the size of the intersection represented by the number of Pfam pairs whose sequence identities were higher than the best 10% alignments of all Pfam domains of the same Pfam ID. Taking into account the underlying distributions of sequence identities between all domain sequences prevented misinterpretation of simpler sequence similarity metrics (e.g., an absolute sequence identity threshold) when different evolutionary rates apply to different protein families. We define structural similarity of a given BGC product pair as the Tanimoto coefficient between the two SMILES strings, using linear-path fingerprints (FP2) from Open Babel [67].

### Sub-cluster analysis of known gene clusters

Sub-clusters with known functions from experimentally characterized gene clusters were manually collected from the literature. Sub-cluster sharing between gene clusters from the training set was calculated using blastp [68]. The minimum requirement used to identify a shared sub-cluster between two BGCs was sharing either 75% of the genes with >45% average sequence identity, 50% of the genes with >50% average sequence identity, or 25% of the genes with 70% identity. To account for different modes of sequence evolution of different sub-cluster types, these values were adjusted with sub-cluster type-specific cutoffs to obtain a good match between genetic similarity and chemical similarity (**Table S3**). The final sub-cluster sharing network was drawn with Cytoscape [69].

### Multimodular NRPS/PKS gene cluster evolution

To study patterns of evolution in multimodular NRPS and PKS gene clusters, a range of features was calculated describing key characteristics of these gene clusters. The first set of features was based on the topologies of intra-BGC domain similarity networks (with protein domains and sequence similarity representing nodes and edges, respectively) and consisted of the average clustering coefficient, average sequence similarity, graph transitivity, number of 2–4 node cliques, number of connected components in a graph with sequence similarity >50%, and average neighbor degree. We also included as features the number of different Pfam domain types in a BGC, the total number of domains in a BGC, the average number of domains per gene, and the averages and standard errors of best-matching pair sequence identities and internal BGC similarity indices. Two evolutionary indices were also added: the internal similarity index and the vertical evolution index. To obtain the internal similarity index of a gene cluster, we calculated for each of its NRPS/PKS domains the *p*-value of its closest blastp match inside the gene cluster, given the distribution of the percent identities of all within-gene-cluster blastp hits of all domains of that domain type in the complete set of gene clusters. The internal similarity index was then calculated from these numbers as the mean of all inverse *p*-values. The same inverse *p*-values were used for plotting the internal domain similarity across gene clusters. The vertical evolution index of a gene cluster was calculated as the average difference between the *p*-value of the top 10 percent identities of a domain's blastp hits to all domains from other gene clusters with the *p*-values of the Lin distances of the gene clusters to the host gene clusters of each of the top 10 hit domains. Consequently, gene clusters with domains with highly similar closest hits to domains in dissimilar gene clusters get a low value, while gene clusters with domains with dissimilar closest hits to domains in similar gene clusters get a high value.

PCA analysis was performed with the aforementioned features as an input. Compound types were assigned using the classifications taken from the primary literature.

## Supporting Information

Figure S1 **The rapid and dynamic evolution of BGCs differs from the evolution of tryptophan operons.** Distributions of the best matching sequence homologs with respect to organism similarity (based on 16S rRNA) for predicted BGCs and tryptophan operons suggest significant differences in the ways they evolve, The distribution of all organism-organism similarities (background organism similarities) is trimodal, which may explain why, similarly, the distributions of the best-matching sequence homologs for predicted BGCs and tryptophan operons are also trimodal.
(PDF)

Figure S2 **Examples of insertions/deletions in BGCs.** Three gene cluster alignments of highly similar BGCs (>70% at the nucleotide level) are shown that are likely to represent relatively recent insertions/deletions in BGCs with functional consequences. In the upper panel, genes that putatively encode one or more sugar moieties have been inserted/deleted from a saccharide biosynthesis gene cluster. In the middle panel, a germacradienol synthase has been replaced by another type of terpene synthase, a pentalenene synthase, as well as an AMP-dependent synthetase. In the lower panel, a gene cluster related to the well-known coelibactin gene cluster from *Saccharopolyspora spinosa* is shown, which has acquired a MSAS polyketide synthase, a cytochrome P450, a carboxamide synthase and a 3-oxoacyl-(ACP) synthase compared to the coelibactin gene cluster from *Streptomyces coelicolor*. These genes are predicted to encode a polyketide moiety that might be attached to the NRP siderophore synthesized by the coelibactin NRPS machinery.
(PDF)

Figure S3 **Phylogeny of ansamycin KS domains.** A FastTree [70] phylogenetic tree of all KS domains from the divergolide, hygromycin, maytansinoid, rubradirin, rifamycin and macbecin gene clusters (which all have an AHBA sub-cluster), was generated with the 10 closest BLAST hits of each domain (outside those to KS domains within the same data set, and after removal of redundancy). Except three, all KS domains cluster monophyletically with other ansamycin KS domains (i.e., other KS domains from gene clusters with an AHBA sub-cluster). Other related KS domains cluster in separate clades.
(PDF)

Figure S4 **Phylogenetic tree of MSA/OSA iterative PKSs.** Maximum likelihood phylogenetic tree (constructed with RAxML [71]) of all known bacterial naphthoic acid/6-methylsalicylic acid/orsellinic acid synthases, with a fungal 6-methylsalicylic acid synthase used as outgroup. The core scaffold/type of the parent BGC in which the MSAS/OSAS sub-cluster resides is denoted with colored squares at the right.
(PDF)

Figure S5 **Similarity between daptomycin and its BGC and other BGCs and their small molecule products.** Node sizes correspond to the number of Pfam domains with sequence identity to one of the daptomycin genes higher than the top 10^th^ percentile of the background Pfam sequence identity distribution, and node colors denote the average sequence identity for such Pfam domain pairs.
(PDF)

Figure S6 **GPL condensation domains clade with daptomycin condensation domains in a phylogenetic tree.** The tree shown was reconstructed using the maximum likelihood method in MEGA [72], after structure-based multiple sequence alignment with PROMALS3D [73]. The C-domain of the GPL starter module clades together with the C-domain of the daptomycin starter module, and the other GPL C-domains clade together with the DCL C-domains from the daptomycin assembly line. A C-domain of the glycopeptide balhimycin (which is closely related to vancomycin) also groups with these domains.
(PDF)

Figure S7 **Mutations in AT and KS domains mapped onto their crystal structures.** **a**, We aligned sequences of AT and KS domains from 4 BGCs (**Fig. 3a**) on a crystal structure of a KS-AT didomain from module 3 of the 6-deoxyerthronolide B synthase (PDB ID: 2QO3) [74]. For each position in the alignment, we assessed sequence variability by calculating entropy based on the amino acid frequencies (color-coded from white to red in chain A; chain B of the homodimer is shown as backbone trace only). **b**, While most of the domain shows a high tendency towards mutations, visual inspection reveals a relatively conserved region at the acetate-binding site of the AT domain. **c**, Mutations in the KS domain, however, appear to cluster in several regions of the structure, including the region around the substrate-binding site (here, denoted by the binding site of the inhibitor cerulenin) and at the homodimer interface. The entropy was not calculated in the regions that fall outside of the Pfam-annotated domains, nor in the indel-rich regions (marked black). The figures were generated using UCSF Chimera [75].
(PDF)

Figure S8 **Evidence for concerted evolution in various PKS and NRPS gene clusters.** Phylogenetic trees of KS/AT and C/A domains, respectively, involved in the biosynthesis of several families of related polyketide or nonribosomal peptide molecules show various degrees of concerted evolution. For example, trees of the AT and KS domains of macrolide biosynthesis enzymes show a high rate of BGC-specific branching (suggestive of concerted evolution), while hardly any such branching is observed in trees of the C and A domains of glycopeptide biosynthetic enzymes. Phylogenetic trees were constructed in MEGA5 [72] with the neighbor-joining method (100 bootstrap replicates), based on alignments of the domain amino acid sequences generated with MUSCLE [63]. For tree construction, all positions containing gaps and missing data were eliminated.
(PDF)

Figure S9 **Clustered heat map of features based on protein sequence alignments and domain-similarity network topologies.** Features include the average number of Pfam domains per gene, means and standard deviations of the clustering coefficient and the network transitivity (see Methods for more details). At least four distinct clusters of BGCs appear from the heat map that have different evolutionary characteristics.
(PDF)

Figure S10 **Domain architectures of all 658 BGCs encoding multimodular PKS and NRPS enzymes.** The domains are colored by the p-value of the homology to their nearest neighbor within the same gene cluster. BGCs that are mostly red contain domains that are highly similar to other domains in the same gene cluster, whereas BGCs that are mostly blue contain domains that are dissimilar from other domains within the same gene cluster.
(PDF)

Figure S11 **Detailed phylogenetic trees of KS and AT domains of polyketide synthases from the rapamycin family.** The tree was reconstructed using the neighbor-joining method in MEGA [72], using 100 bootstrap replicates.
(PDF)

Table S1 **Overview of evolutionary events detected between alignments of gene cluster pairs sharing at least three matching 1 kb-sized blocks in alignments with thresholds of >70% identity (top) or >80% identity (bottom).** The numbers of observed indels, duplications and rearrangements are given for BGCs of several sizes classes: 1–10 kb, 11–20 kb, 21–30 kb, 31–40 kb and 40+ kb, or in cumulative combinations of these size classes (>10 kb, >20 kb, >30 kb, >40 kb).
(XLSX)

Table S2 **Results from the phylogenetic profiling analysis at three different cross-correlation cutoffs.** The first and second column of each table show a number of co-evolving and non-coevolving motifs, followed by p-values from a Chi^2^-test (in which the first two numbers were assumed to be equally distributed), a string of Pfam IDs that constitute a motif, and their description.
(XLSX)

Table S3 **Sub-cluster type-specific cut-offs to determine sub-cluster sharing between BGCs.** Suitable cut-offs were determined by a manual comparison of known sub-clusters to their corresponding known chemical moieties, and cut-offs were set at those sequence identities where the chemistry produced by the enzymes encoded by the sub-clusters was (nearly) identical.
(XLSX)

Data S1 **Python script and associated data files that were used to identify insertions/deletions, duplications and rearrangements in homologous gene clusters.**
(ZIP)
