# Correction: A Systematic Computational Analysis of Biosynthetic Gene Cluster Evolution: Lessons for Engineering Biosynthesis

**DOI:** 10.1371/journal.pcbi.1004767

**Published:** 2016-03-08

**Authors:** The PLOS Computational Biology Staff

The authors have discovered a bug in the code for one of their analyses that renders a minor conclusion incorrect. There are some errors in Fig 5 and the corresponding S10 Fig.

**Fig 5:**
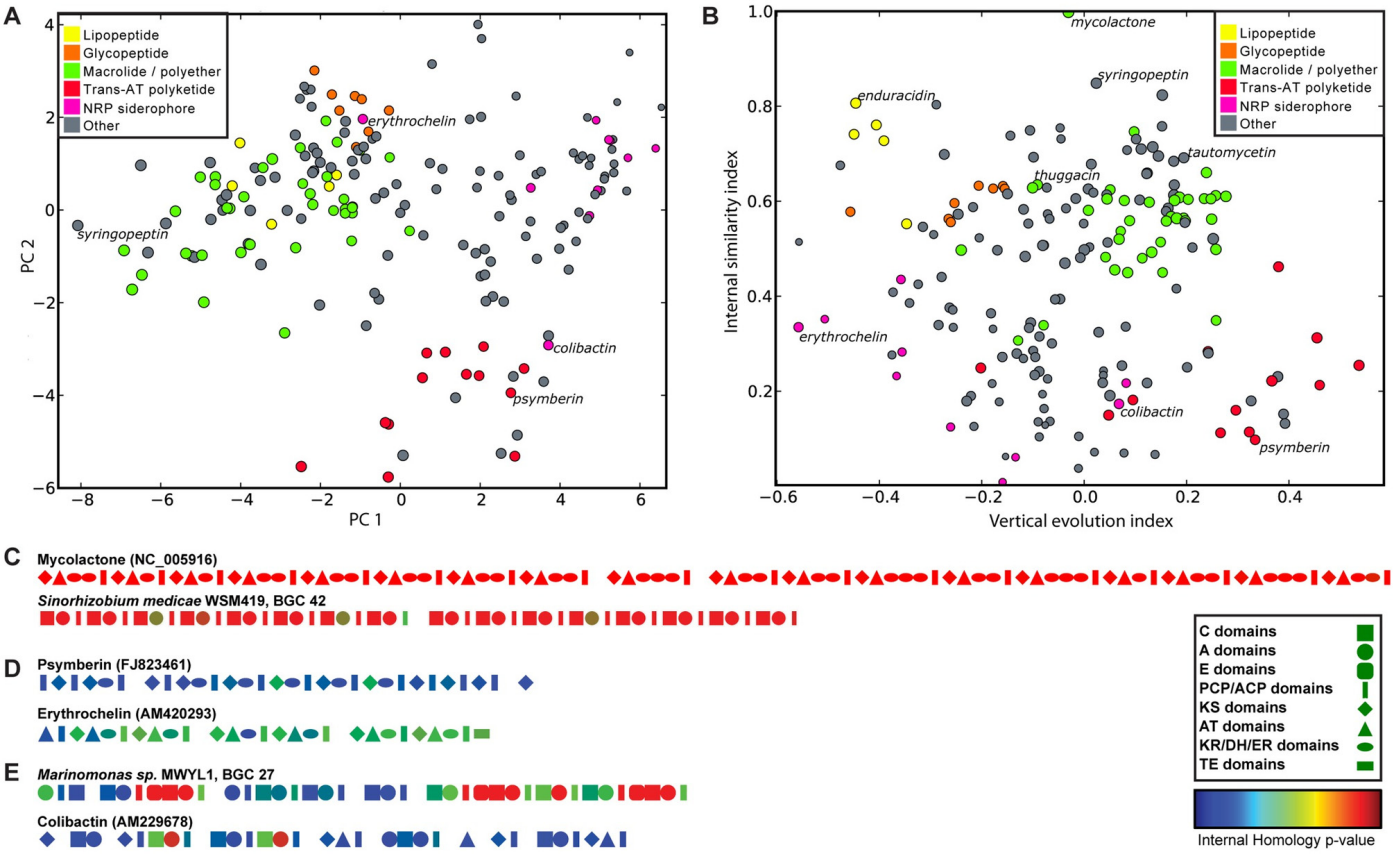
Diverse and distinct modes of evolution for PKS and NRPS BGCs. a, Scatter plot showing the first two principal components resulting from a PCA analysis of different evolutionary characteristics of BGCs encoding different classes of NRPs and PKs. The first two principal components describe 63% of the variance. BGCs encoding members of the same family (e.g., lipopeptides, glycopeptides or macrolides) tend to cluster together, suggesting that their family members evolve in similar ways, while different families cluster apart from each other, suggesting distinct modes of evolution. Colors indicate distinct classes of BGCs. b, Scatter plot showing two features of BGCs—internal similarity index and vertical evolution index—that, of the 25 measured features, underly most of the variation. The internal similarity index indicates how similar domains in a BGC are to other domains within the same BGC. The vertical evolution index indicates how closely related a BGC is to the BGCs harboring the closest relatives of its constituent domains (see Methods for more details). Colors indicate distinct classes of BGCs, as in panel a. c-e, Domain architecture plots of PKSs and NRPSs show distinct modes of evolution: c, Internal duplication with concerted evolution; d, domain swapping with other BGCs; and e, mixed evolution. Geometric shapes indicate domain types (see legend); domain colors indicate the internal homology p-value of each domain to its closest relative within the same gene cluster, within the total distribution of all similarities between domains of the same type in the entire data set: hence, domains colored red are most similar, while domains colored blue are most dissimilar.

In the main text, the following sentences should be disregarded:

“Secondly, we sometimes observed gradients of the internal homology p-values from the N- to C-termini of large synthases, suggesting that some gene clusters evolve to encode the synthesis of larger molecules by iterative duplication of their most N-terminal module, would have the effect of extending an intermediate NRP or PK by the addition of a new starter unit.”

and

“Also, evolutionary strategies towards generating larger and more complex compounds could be mimicked by N-terminally extending certain types of NRPS/PKS gene clusters by duplicating and then carefully modifying the first assembly-line module.”

## Supporting Information

S10 Fig Domain architectures of all 658 BGCs encoding multimodular PKS and NRPS enzymes. The domains are colored by the p-value of the homology to their nearest neighbor within the same gene cluster. BGCs that are mostly red contain domains that are highly similar to other domains in the same gene cluster, whereas BGCs that are mostly blue contain domains that are dissimilar from other domains within the same gene cluster.
(TIF)
