# Supplementary figures and images for: A Systematic Computational Analysis of Biosynthetic Gene Cluster Evolution: Lessons for Engineering Biosynthesis
Source: PLoS Comput Biol. 2014 Dec 4;10(12):e1004016. doi: 10.1371/journal.pcbi.1004016 (PMC4256081; doi:10.1371/journal.pcbi.1004016)

Figure S1

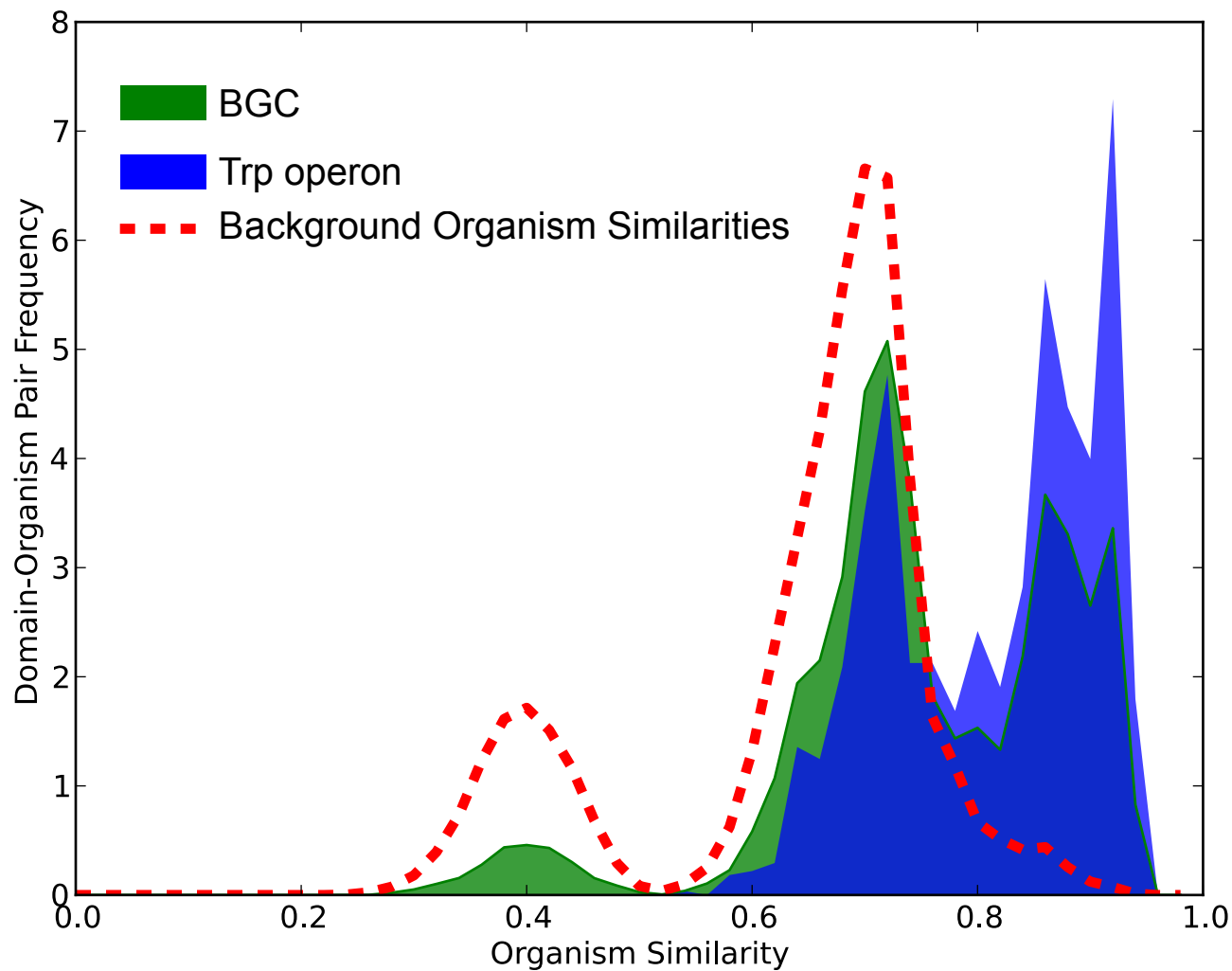

Supplement: Figure S1 — The rapid and dynamic evolution of BGCs differs from the evolution of tryptophan operons. Distributions of the best matching sequence homologs with respect to organism similarity (based on 16S rRNA) for predicted BGCs and tryptophan operons suggest significant differences in the ways they evolve, The distribution of all organism-organism similarities (background organism similarities) is trimodal, which may explain why, similarly, the distributions of the best-matching sequence homologs for predicted BGCs and tryptophan operons are also trimodal. (PDF) [file pcbi.1004016.s001.pdf]

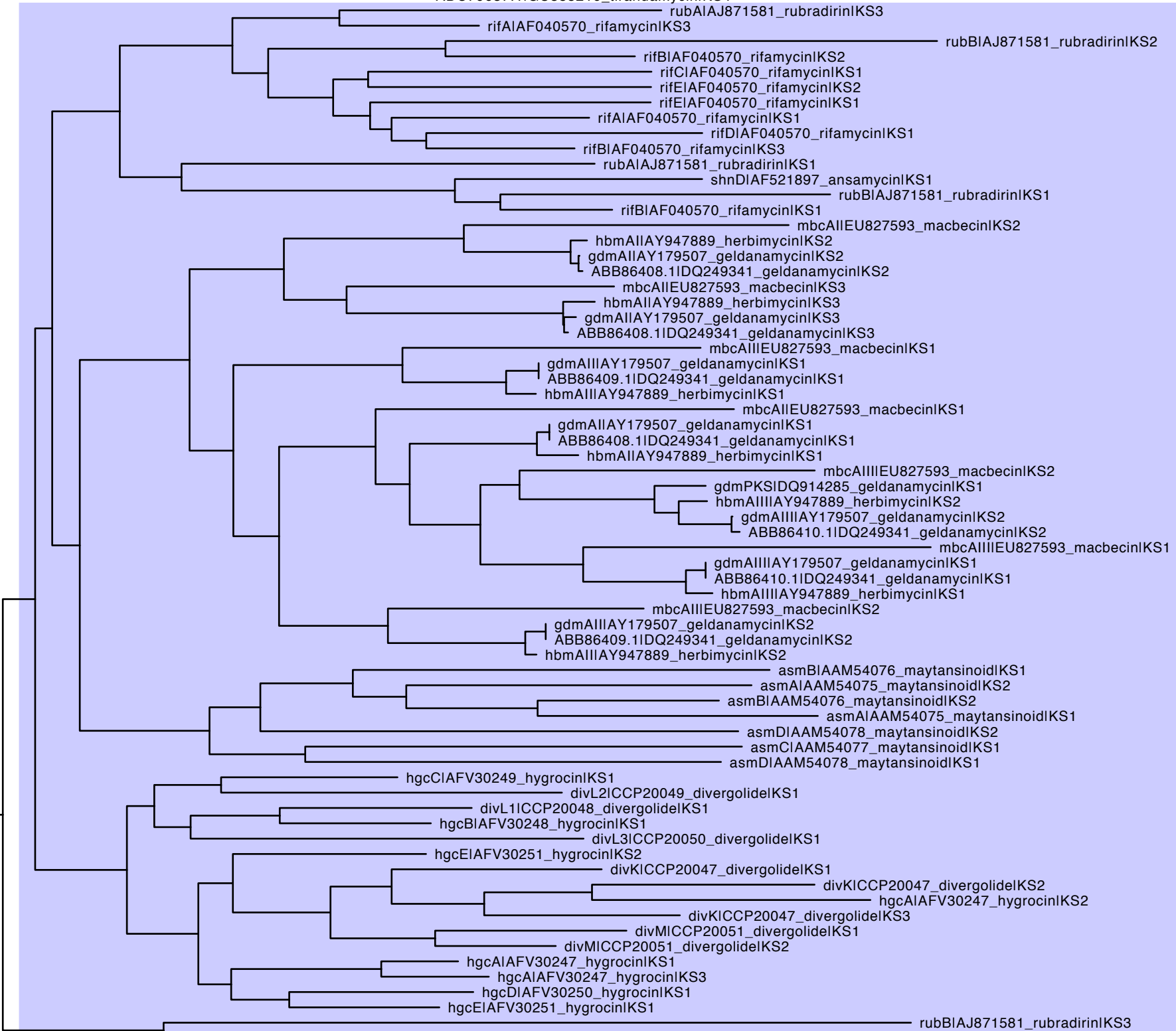

Ansamycin KS domains

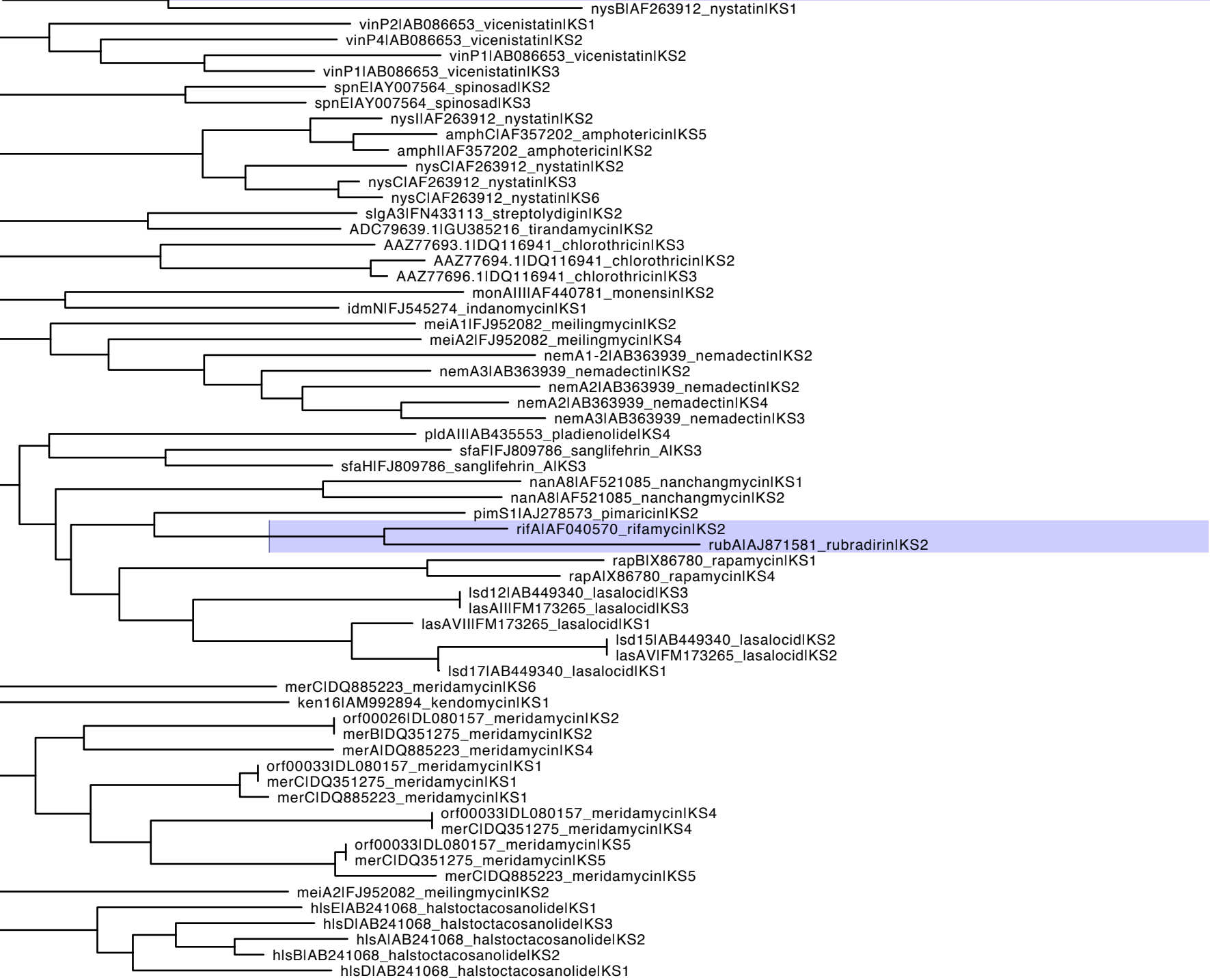

Supplement: Figure S3 — Phylogeny of ansamycin KS domains. A FastTree [70] phylogenetic tree of all KS domains from the divergolide, hygromycin, maytansinoid, rubradirin, rifamycin and macbecin gene clusters (which all have an AHBA sub-cluster), was generated with the 10 closest BLAST hits of each domain (outside those to KS domains within the same data set, and after removal of redundancy). Except three, all KS domains cluster monophyletically with other ansamycin KS domains (i.e., other KS domains from gene clusters with an AHBA sub-cluster). Other related KS domains cluster in separate clades. (PDF) [file pcbi.1004016.s003.pdf]

Figure S5

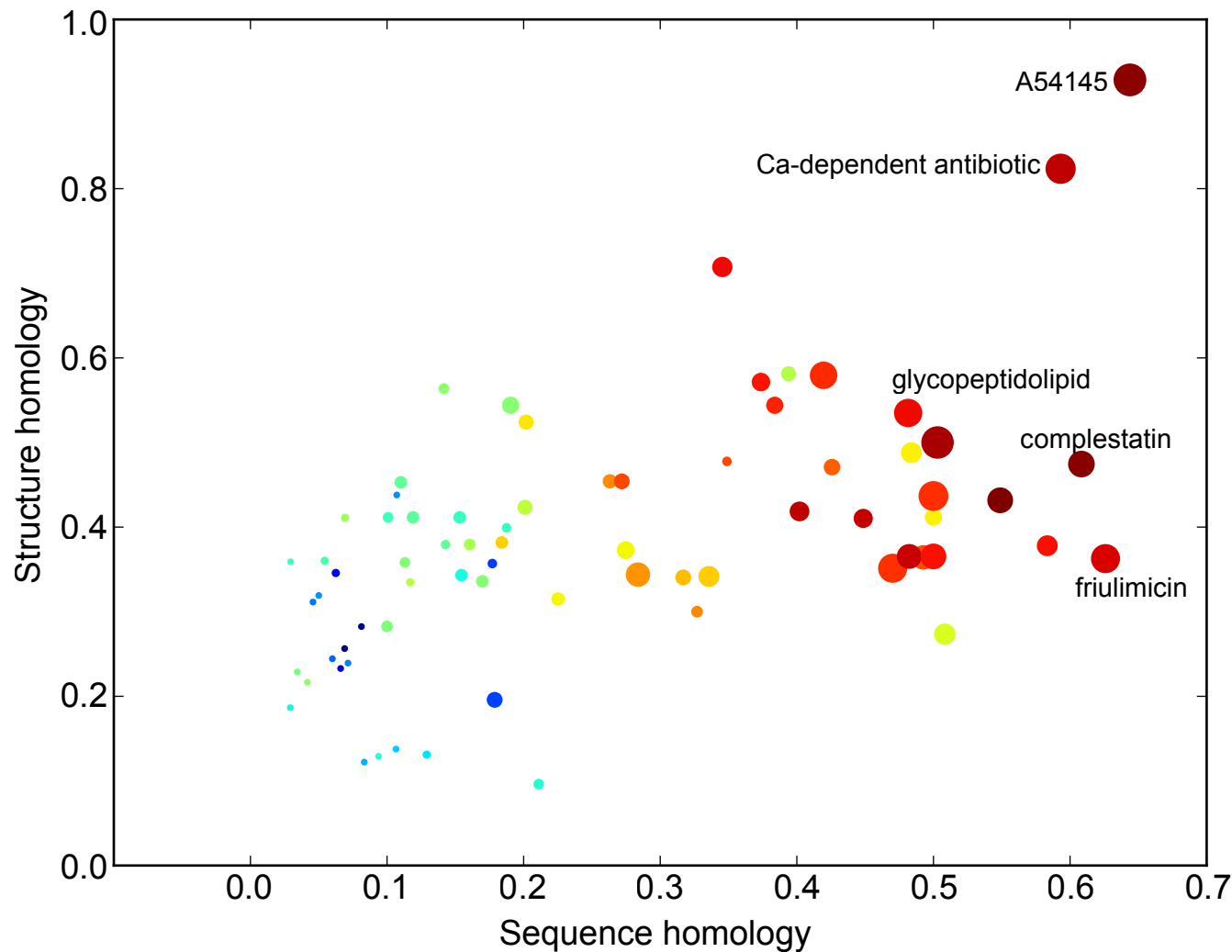

Supplement: Figure S5 — Similarity between daptomycin and its BGC and other BGCs and their small molecule products. Node sizes correspond to the number of Pfam domains with sequence identity to one of the daptomycin genes higher than the top 10th percentile of the background Pfam sequence identity distribution, and node colors denote the average sequence identity for such Pfam domain pairs. (PDF) [file pcbi.1004016.s005.pdf]

Figure S6

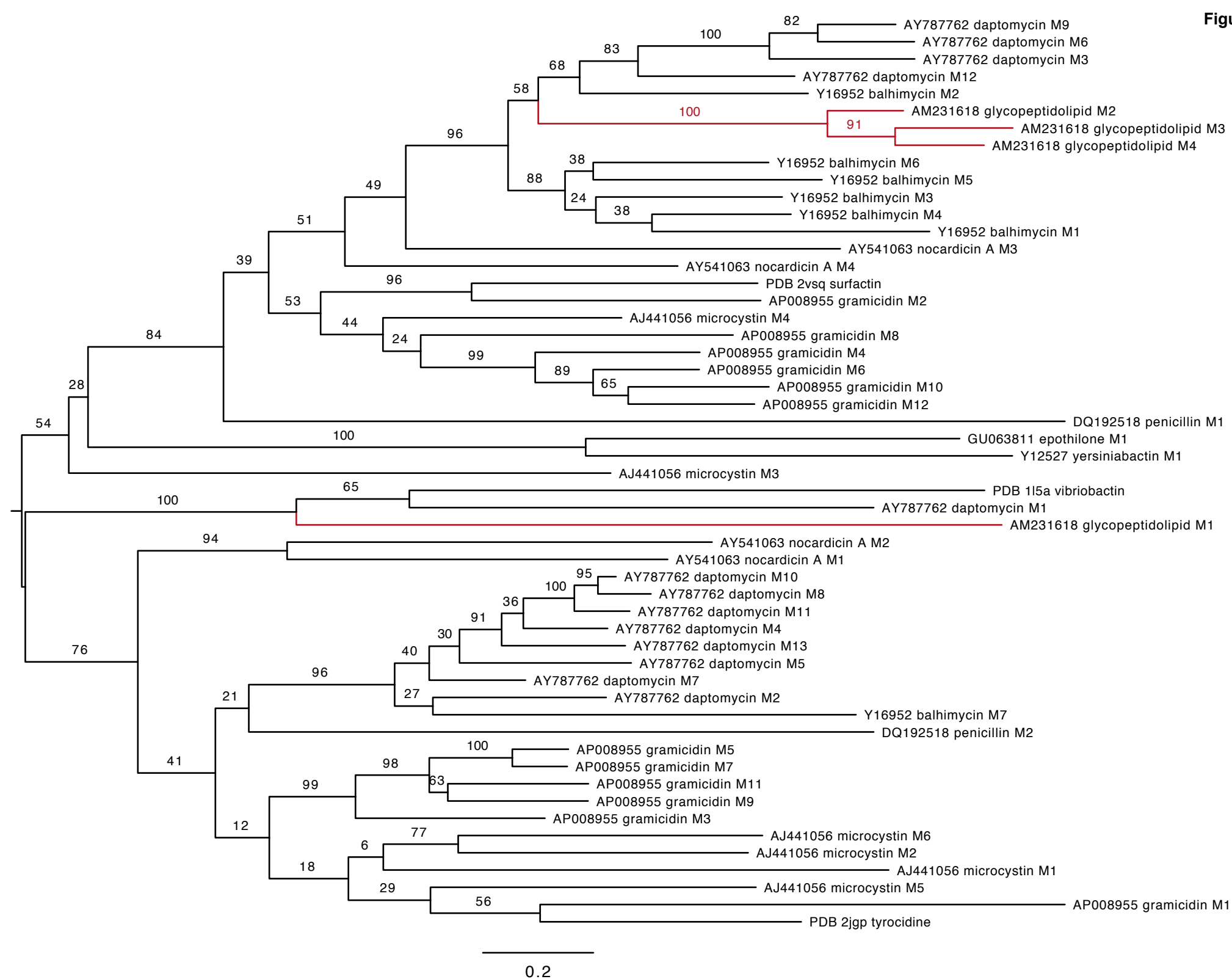

Supplement: Figure S6 — GPL condensation domains clade with daptomycin condensation domains in a phylogenetic tree. The tree shown was reconstructed using the maximum likelihood method in MEGA [72], after structure-based multiple sequence alignment with PROMALS3D [73]. The C-domain of the GPL starter module clades together with the C-domain of the daptomycin starter module, and the other GPL C-domains clade together with the DCL C-domains from the daptomycin assembly line. A C-domain of the glycopeptide balhimycin (which is closely related to vancomycin) also groups with these domains. (PDF) [file pcbi.1004016.s006.pdf]

A

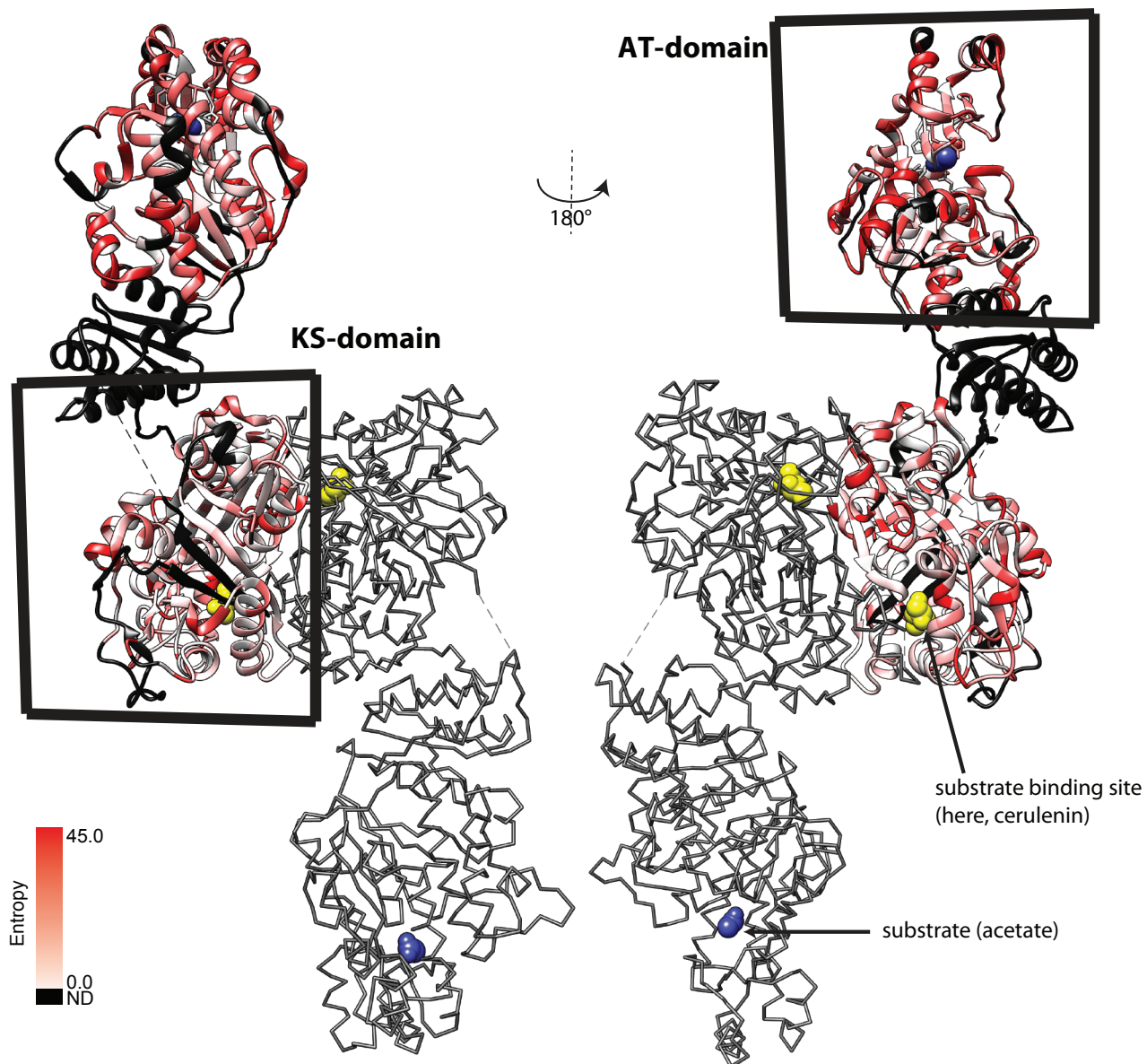

B

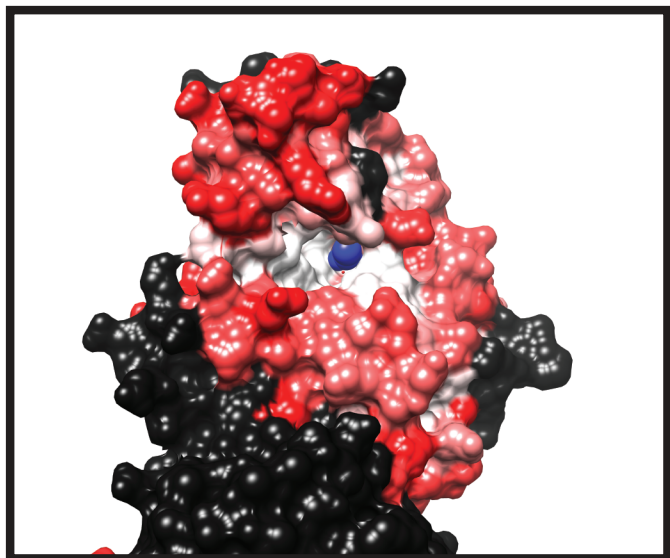

C

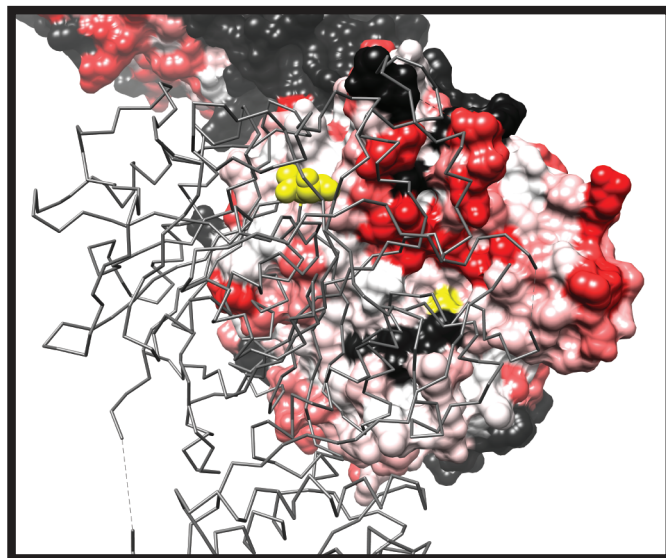

Supplement: Figure S7 — Mutations in AT and KS domains mapped onto their crystal structures. a, We aligned sequences of AT and KS domains from 4 BGCs ( Fig. 3a ) on a crystal structure of a KS-AT didomain from module 3 of the 6-deoxyerthronolide B synthase (PDB ID: 2QO3) [74]. For each position in the alignment, we assessed sequence variability by calculating entropy based on the amino acid frequencies (color-coded from white to red in chain A; chain B of the homodimer is shown as backbone trace only). b, While most of the domain shows a high tendency towards mutations, visual inspection reveals a relatively conserved region at the acetate-binding site of the AT domain. c, Mutations in the KS domain, however, appear to cluster in several regions of the structure, including the region around the substrate-binding site (here, denoted by the binding site of the inhibitor cerulenin) and at the homodimer interface. The entropy was not calculated in the regions that fall outside of the Pfam-annotated domains, nor in the indel-rich regions (marked black). The figures were generated using UCSF Chimera [75]. (PDF) [file pcbi.1004016.s007.pdf]

## Ansamycins

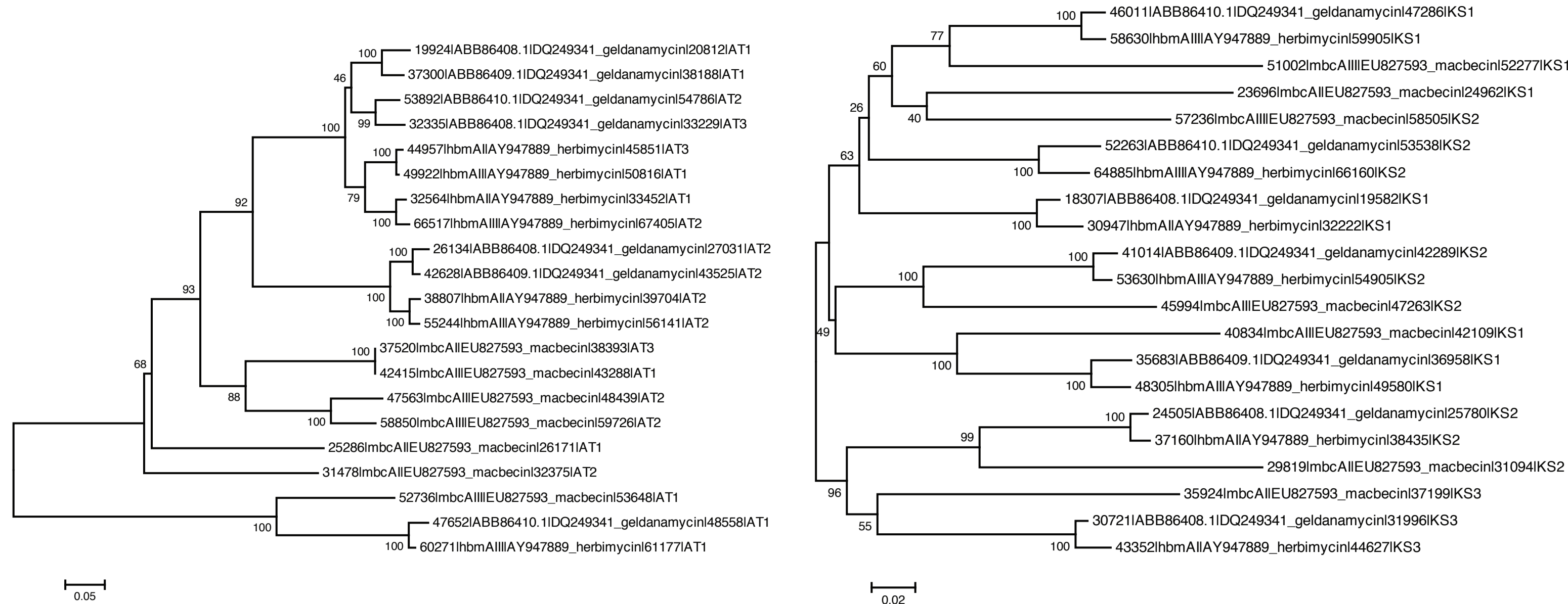

## Macrolides

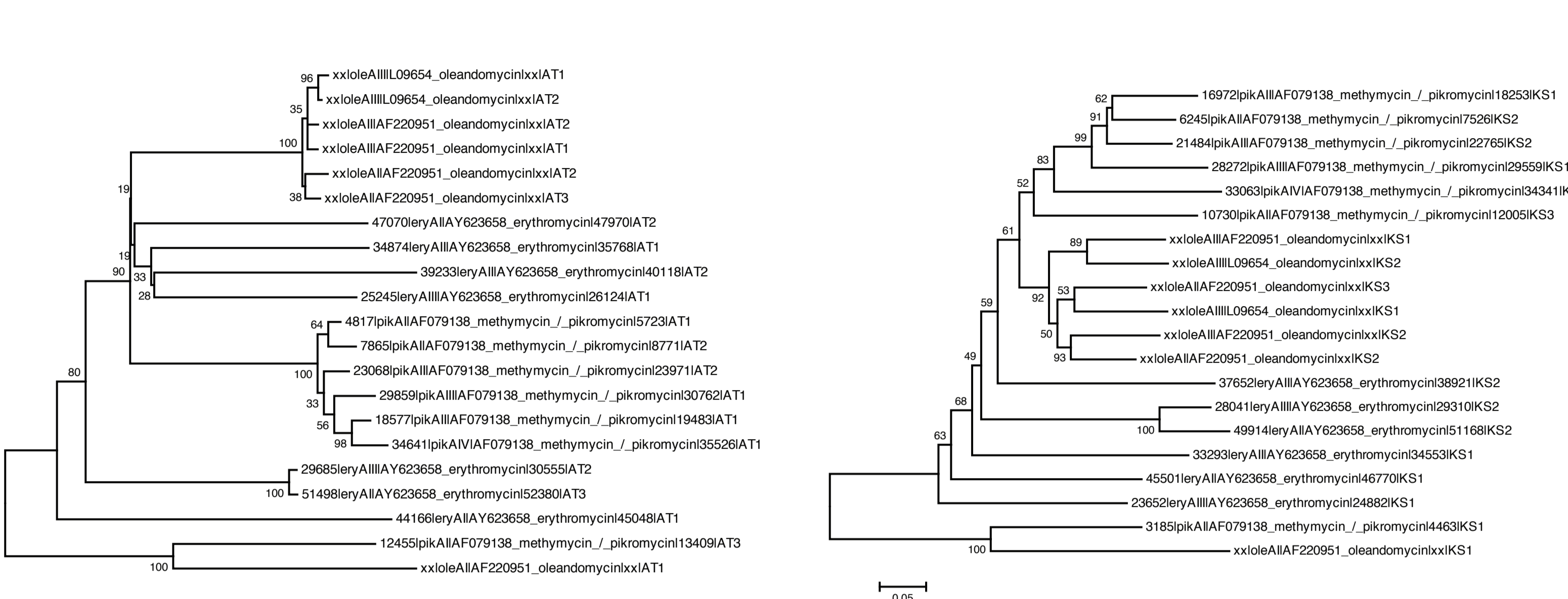

## Antifungal polyenes

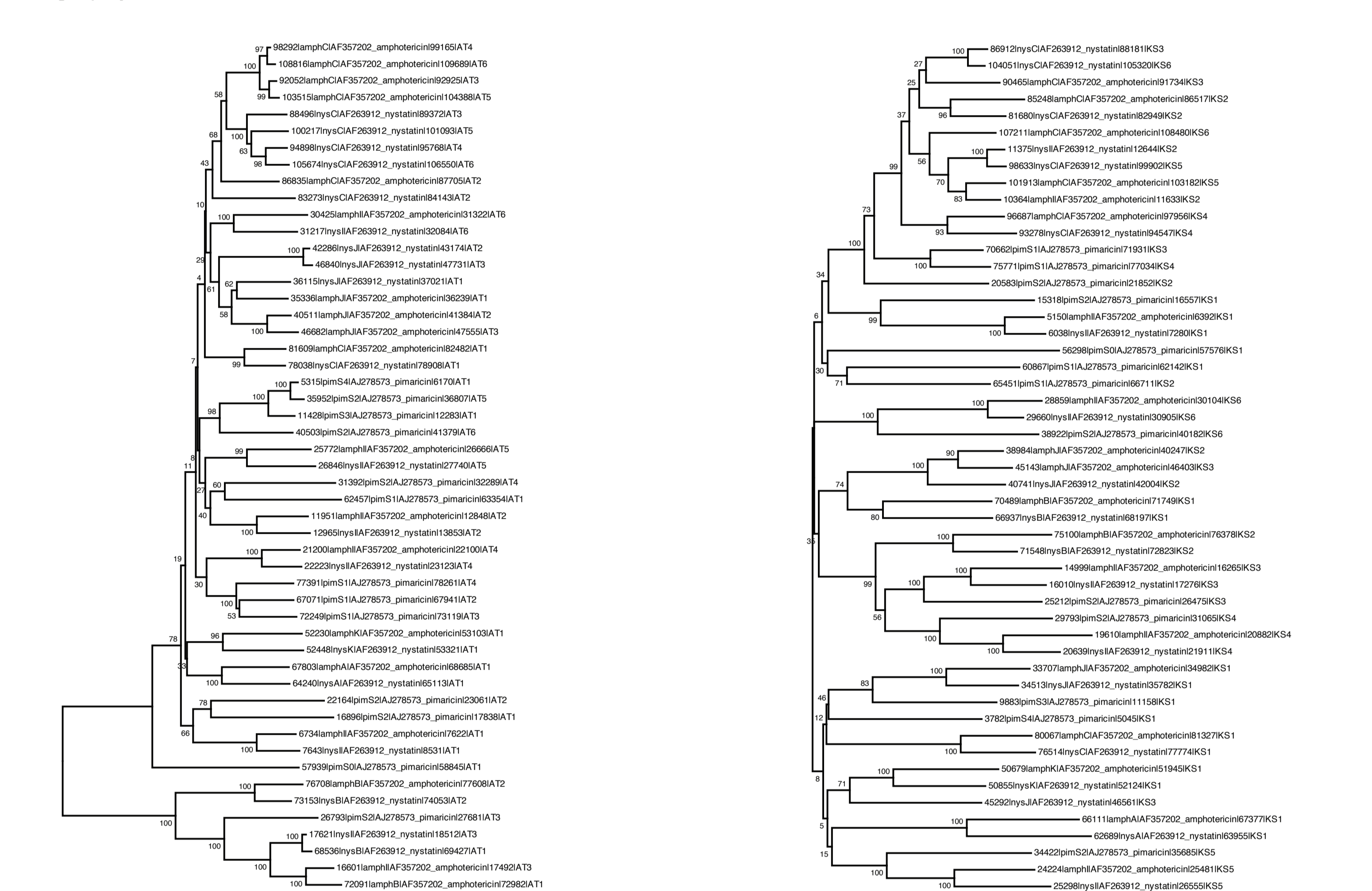

## Glycopeptides

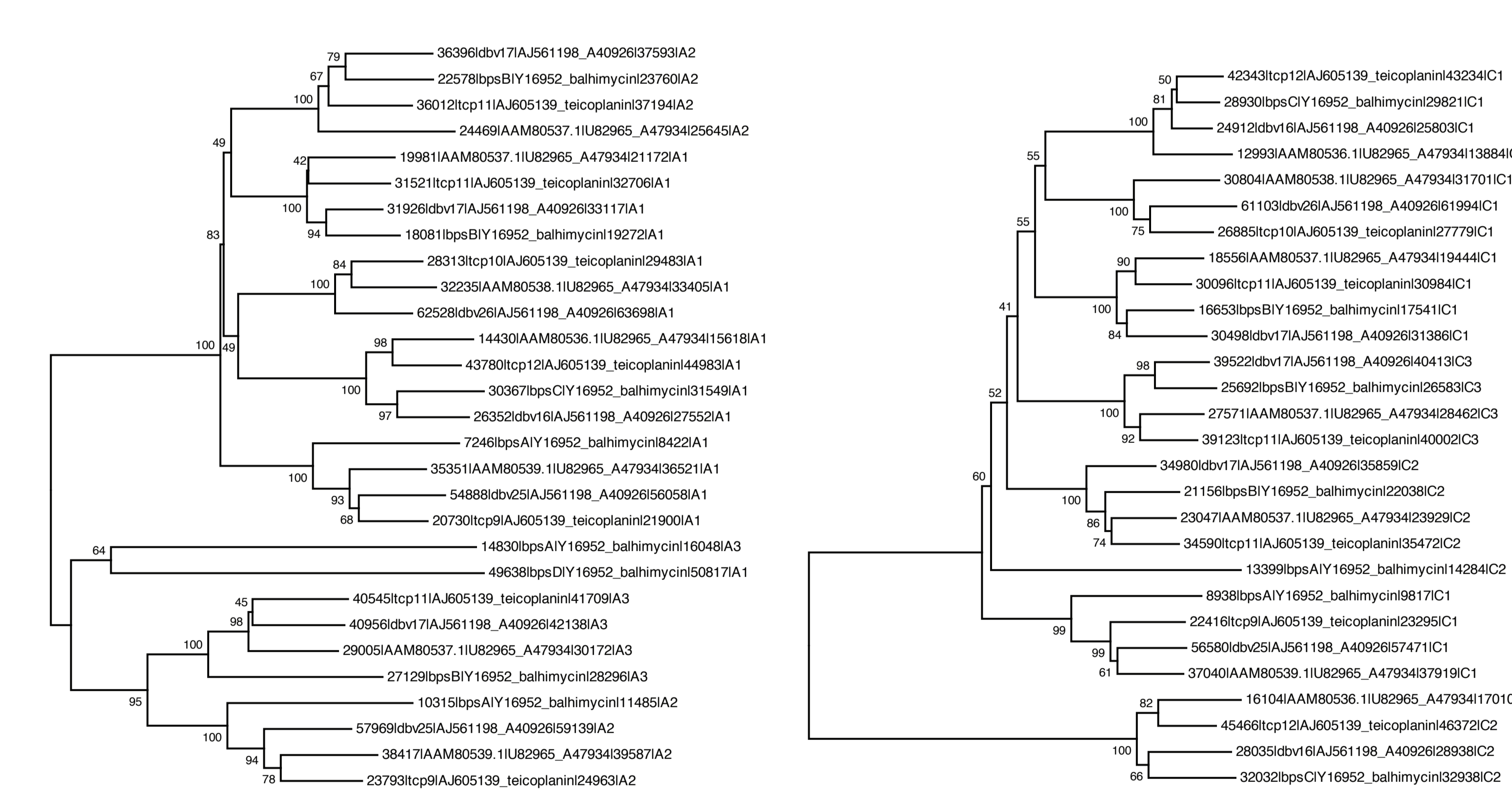

## Calcium-dependent lipopeptides

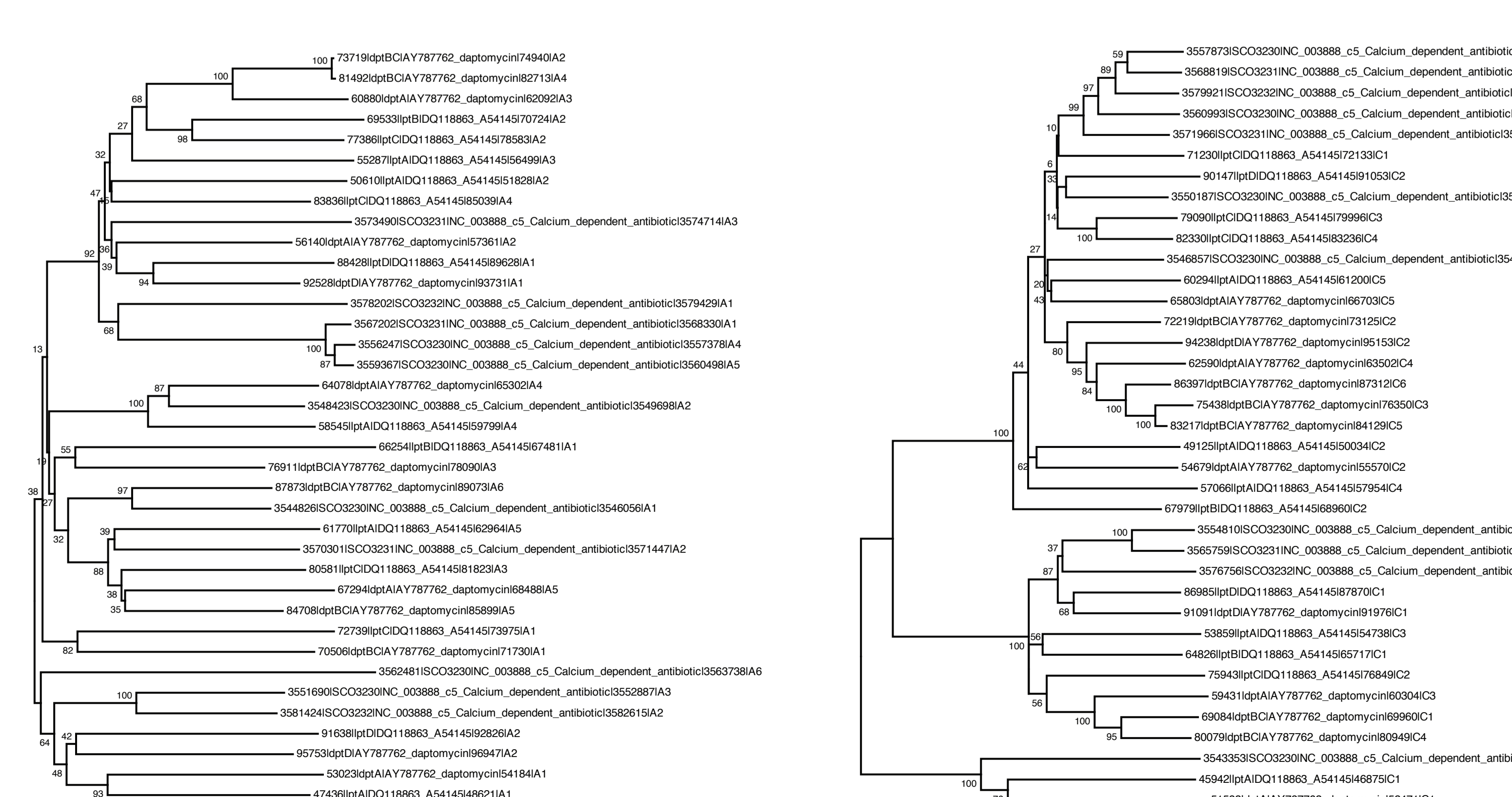

Supplement: Figure S8 — Evidence for concerted evolution in various PKS and NRPS gene clusters. Phylogenetic trees of KS/AT and C/A domains, respectively, involved in the biosynthesis of several families of related polyketide or nonribosomal peptide molecules show various degrees of concerted evolution. For example, trees of the AT and KS domains of macrolide biosynthesis enzymes show a high rate of BGC-specific branching (suggestive of concerted evolution), while hardly any such branching is observed in trees of the C and A domains of glycopeptide biosynthetic enzymes. Phylogenetic trees were constructed in MEGA5 [72] with the neighbor-joining method (100 bootstrap replicates), based on alignments of the domain amino acid sequences generated with MUSCLE [63]. For tree construction, all positions containing gaps and missing data were eliminated. (PDF) [file pcbi.1004016.s008.pdf]

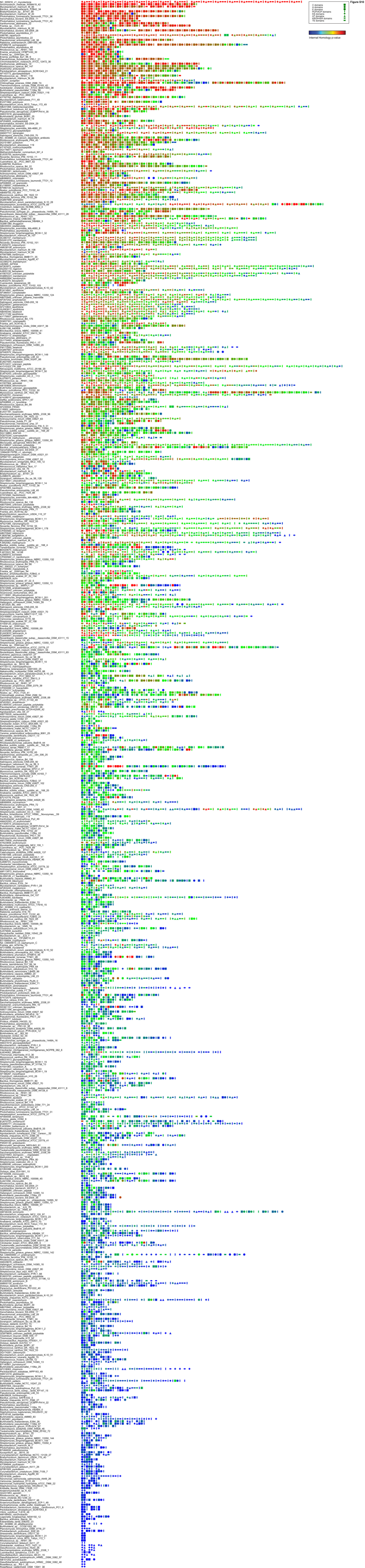

Supplement: Figure S10 — Domain architectures of all 658 BGCs encoding multimodular PKS and NRPS enzymes. The domains are colored by the p-value of the homology to their nearest neighbor within the same gene cluster. BGCs that are mostly red contain domains that are highly similar to other domains in the same gene cluster, whereas BGCs that are mostly blue contain domains that are dissimilar from other domains within the same gene cluster. (PDF) [file pcbi.1004016.s010.pdf]

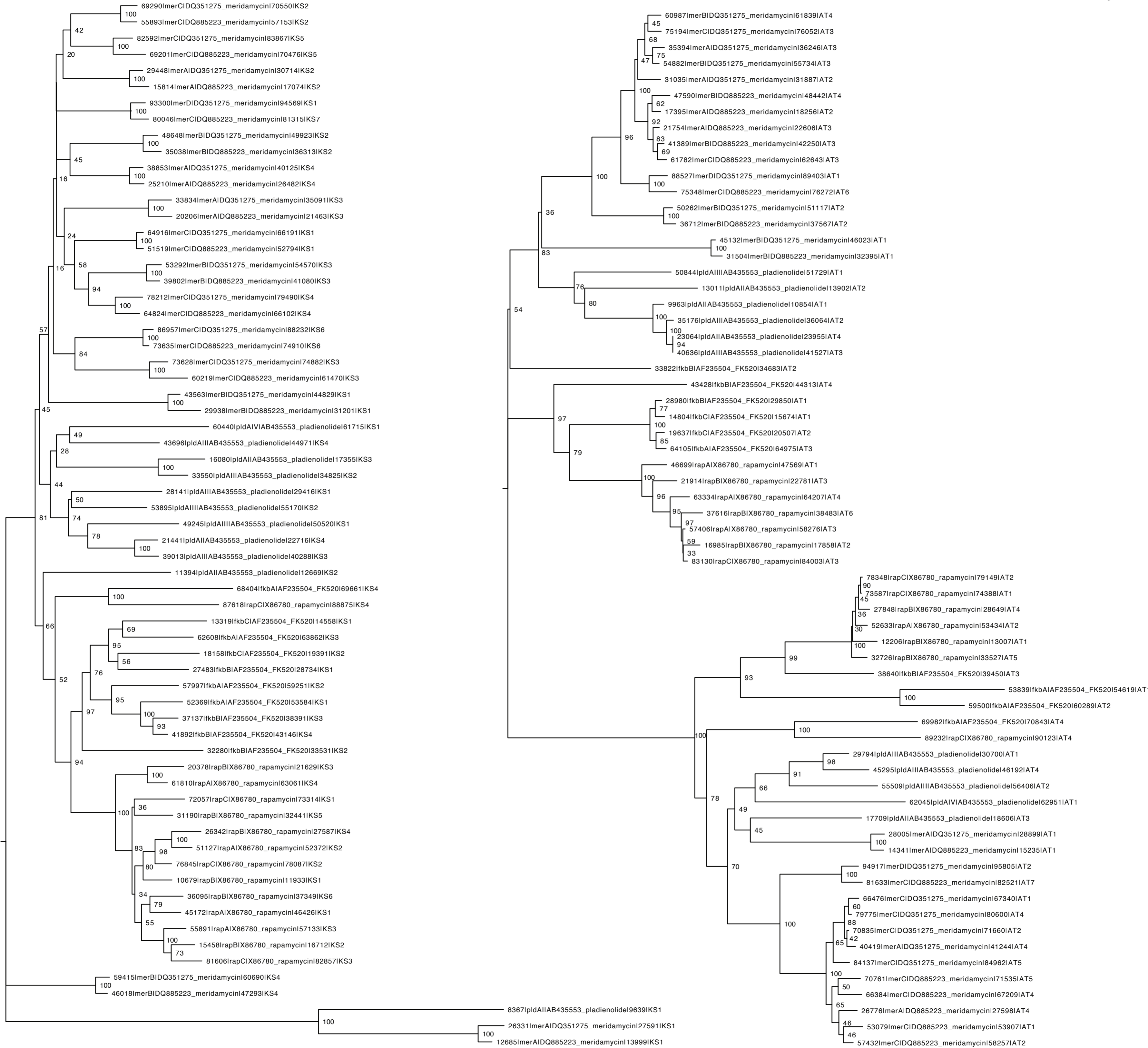

0.06

0.1

Supplement: Figure S11 — Detailed phylogenetic trees of KS and AT domains of polyketide synthases from the rapamycin family. The tree was reconstructed using the neighbor-joining method in MEGA [72], using 100 bootstrap replicates. (PDF) [file pcbi.1004016.s011.pdf]
