# Supplementary material for: A Systematic Computational Analysis of Biosynthetic Gene Cluster Evolution: Lessons for Engineering Biosynthesis
Source: PLoS Comput Biol. 2014 Dec 4;10(12):e1004016. doi: 10.1371/journal.pcbi.1004016 (PMC4256081; doi:10.1371/journal.pcbi.1004016)
Supplement: Figure S2 — Examples of insertions/deletions in BGCs. Three gene cluster alignments of highly similar BGCs (>70% at the nucleotide level) are shown that are likely to represent relatively recent insertions/deletions in BGCs with functional consequences. In the upper panel, genes that putatively encode one or more sugar moieties have been inserted/deleted from a saccharide biosynthesis gene cluster. In the middle panel, a germacradienol synthase has been replaced by another type of terpene synthase, a pentalenene synthase, as well as an AMP-dependent synthetase. In the lower panel, a gene cluster related to the well-known coelibactin gene cluster from Saccharopolyspora spinosa is shown, which has acquired a MSAS polyketide synthase, a cytochrome P450, a carboxamide synthase and a 3-oxoacyl-(ACP) synthase compared to the coelibactin gene cluster from Streptomyces coelicolor. These genes are predicted to encode a polyketide moiety that might be attached to the NRP siderophore synthesized by the coelibactin NRPS machinery. (PDF) [file pcbi.1004016.s002.pdf]

Figure S2

***Escherichia coli* MS 107-1**
HMPREF9345_01709 - 017041
35 kb

***Shigella sonnei* Ss046**
SSON_2098-2114
20 kb

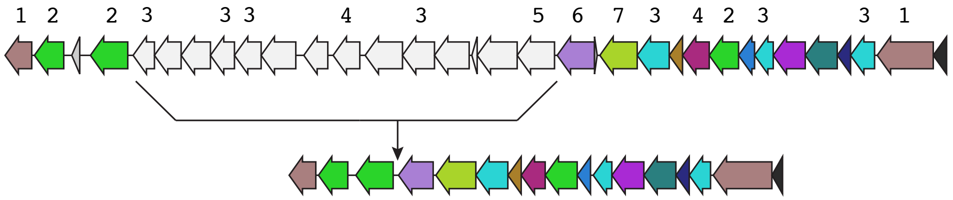

1 **chain-length determinant protein**
2 **sugar dehydrogenase / dehydratase**
3 **glycosyltransferase**
4 **sugar epimerase**
5 **phosphotransferase**
6 **phosphoglucomutase**
7 **sugar isomerase**

***Streptomyces clavuligerus* ATCC 27064**
SCLAV_0153-0165
17 kb

***Streptomyces violaceusniger* Tu 4113**
Strvi_5742-5755
19 kb

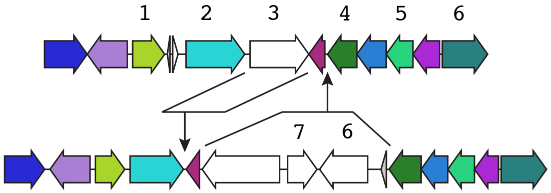

1 **phosphotransferase**
2 **amidase**
3 **germacradienol synthase**
4 **acetyltransferase**
5 **enoyl-CoA hydratase/isomerase**
6 **AMP-dependent synthetase**
7 **pentalenene synthase**

***Saccharopolyspora spinosa* NRRL 18395**
SspiN1_010100010924-010100011009
37 kb

***Streptomyces coelicolor* A3(2)**
SCO7690-7677 (coelibactin gene cluster)
24 kb

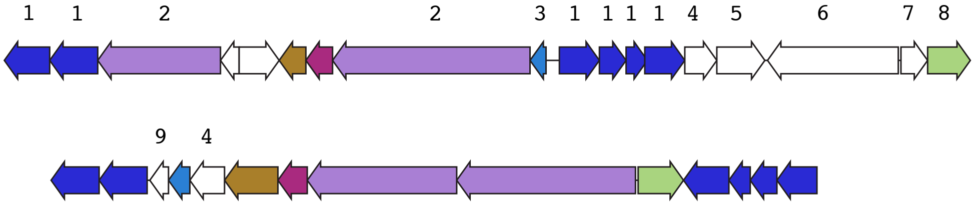

1 **transporter protein**
2 **nonribosomal peptide synthetase**
3 **thioesterase**
4 **cytochrome P450**
5 **carboxamide synthase**
6 **MSAS polyketide synthase**
7 **3-oxoacyl-(ACP) synthase**
8 **AMP-dependent ligase**
9 **methyltransferase**
