# Supplementary material for: A Systematic Computational Analysis of Biosynthetic Gene Cluster Evolution: Lessons for Engineering Biosynthesis
Source: PLoS Comput Biol. 2014 Dec 4;10(12):e1004016. doi: 10.1371/journal.pcbi.1004016 (PMC4256081; doi:10.1371/journal.pcbi.1004016)
Supplement: Figure S4 — Phylogenetic tree of MSA/OSA iterative PKSs. Maximum likelihood phylogenetic tree (constructed with RAxML [71]) of all known bacterial naphthoic acid/6-methylsalicylic acid/orsellinic acid synthases, with a fungal 6-methylsalicylic acid synthase used as outgroup. The core scaffold/type of the parent BGC in which the MSAS/OSAS sub-cluster resides is denoted with colored squares at the right. (PDF) [file pcbi.1004016.s004.pdf]

Figure S4

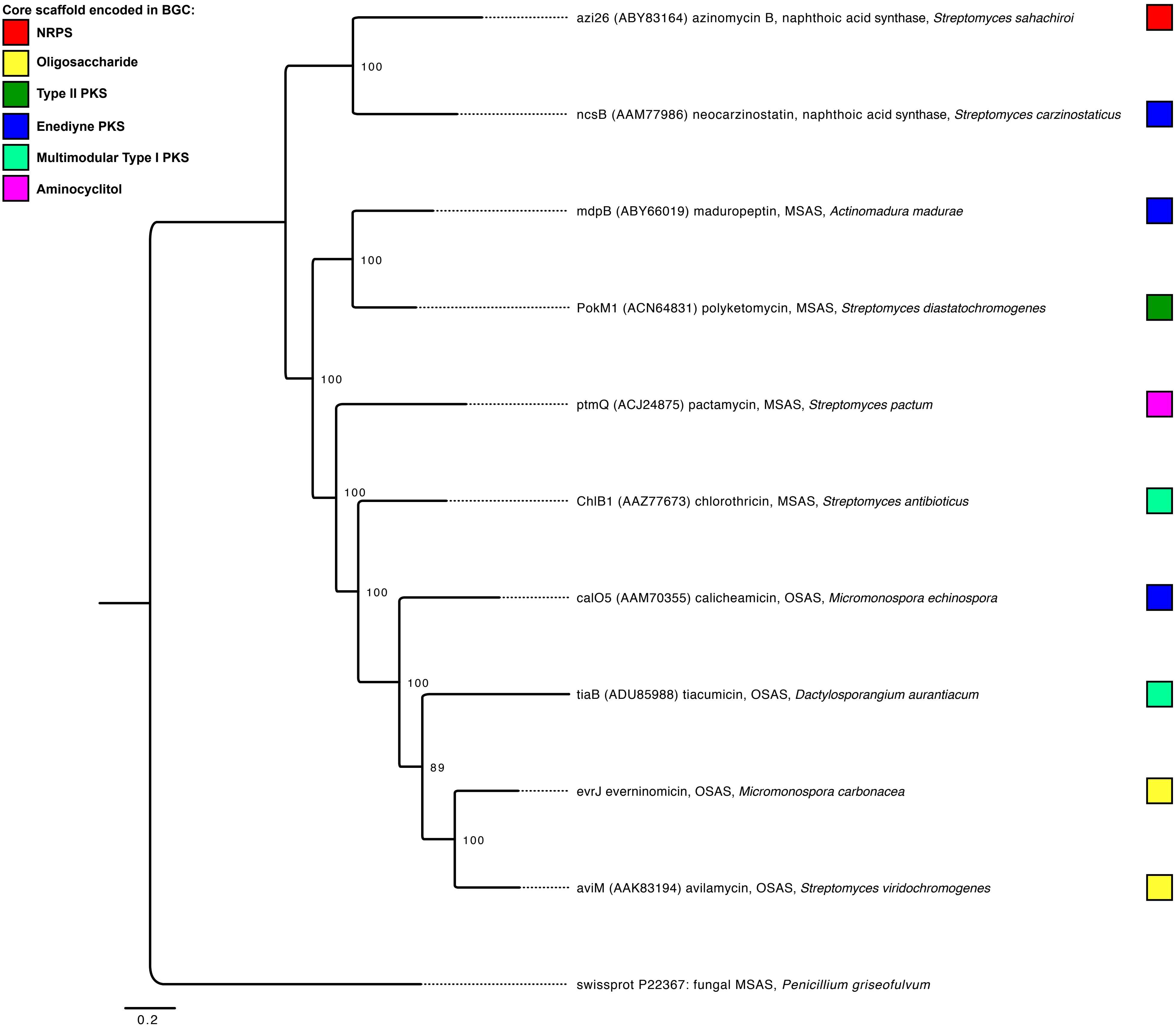
Core scaffold encoded in BGC:
NRPS
Oligosaccharide
Type II PKS
Enediyne PKS
Multimodular Type I PKS
Aminocyclitol
azi26 (ABY83164) azinomycin B, naphthoic acid synthase, Streptomyces sahachiroi
ncsB (AAM77986) neocarzinostatin, naphthoic acid synthase, Streptomyces carzinostaticus
mdpB (ABY66019) maduropeptin, MSAS, Actinomadura madurae
PokM1 (ACN64831) polyketomycin, MSAS, Streptomyces diastatochromogenes
ptmQ (ACJ24875) pactamycin, MSAS, Streptomyces pactum
ChlB1 (AAZ77673) chlorothricin, MSAS, Streptomyces antibioticus
calO5 (AAM70355) calicheamicin, OSAS, Micromonospora echinospora
tiaB (ADU85988) tiacumicin, OSAS, Dactylosporangium aurantiacum
evrJ everninomicin, OSAS, Micromonospora carbonacea
aviM (AAK83194) avilamycin, OSAS, Streptomyces viridochromogenes
swissprot P22367: fungal MSAS, Penicillium griseofulvum
100
100
100
100
100
100
89
100
0.2
