# Supplementary material for: A Systematic Computational Analysis of Biosynthetic Gene Cluster Evolution: Lessons for Engineering Biosynthesis
Source: PLoS Comput Biol. 2014 Dec 4;10(12):e1004016. doi: 10.1371/journal.pcbi.1004016 (PMC4256081; doi:10.1371/journal.pcbi.1004016)
Supplement: Figure S9 — Clustered heat map of features based on protein sequence alignments and domain-similarity network topologies. Features include the average number of Pfam domains per gene, means and standard deviations of the clustering coefficient and the network transitivity (see Methods for more details). At least four distinct clusters of BGCs appear from the heat map that have different evolutionary characteristics. (PDF) [file pcbi.1004016.s009.pdf]

Figure S9

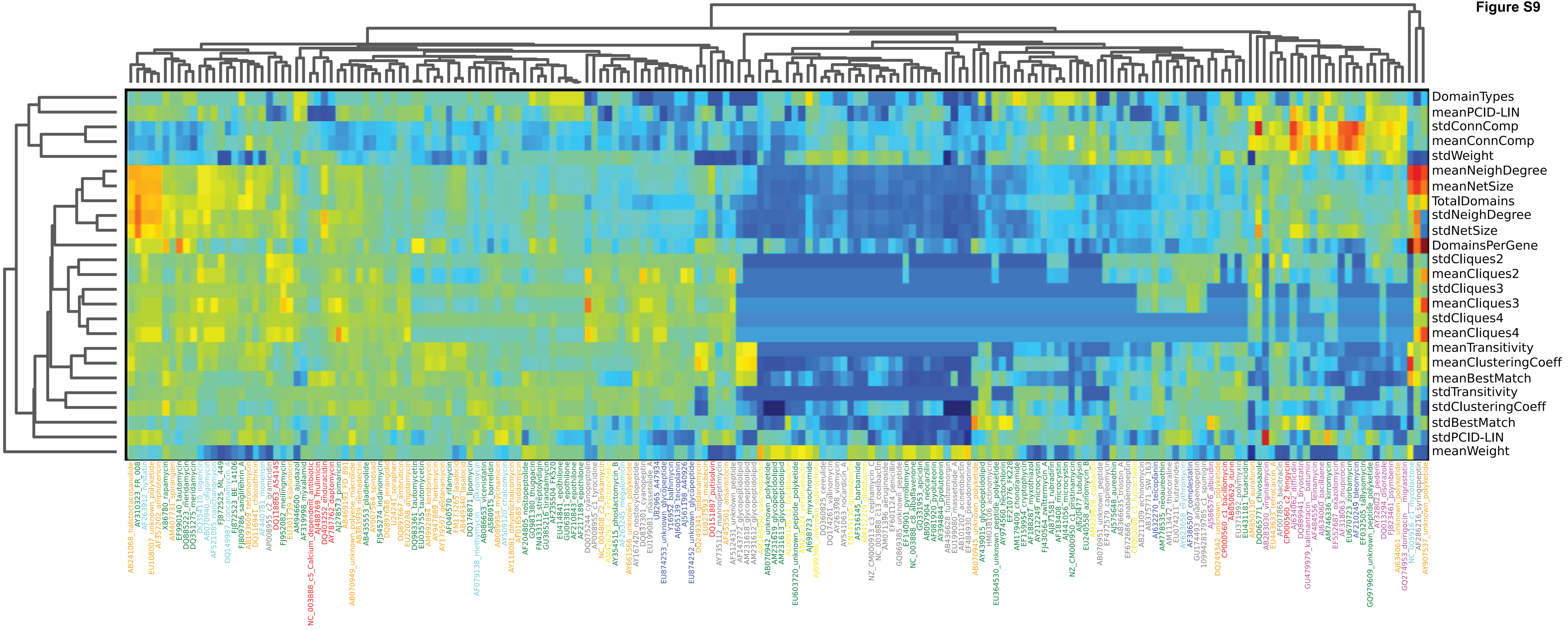
DomainTypes
meanPCID-LIN
stdConnComp
meanConnComp
stdWeight
meanNeighDegree
meanNetSize
TotalDomains
stdNeighDegree
stdNetSize
DomainsPerGene
stdCliques2
meanCliques2
stdCliques3
meanCliques3
stdCliques4
meanCliques4
meanTransitivity
meanClusteringCoeff
meanBestMatch
stdTransitivity
stdClusteringCoeff
stdBestMatch
stdPCID-LIN
meanWeight
